# *Lactiplantibacillus plantarum* ^WJL^ ameliorates chronic kidney disease by inhibiting fibroblast growth factor 21 adaptive stress response via low protein diet

**DOI:** 10.1080/19490976.2026.2696622

**Published:** 2026-07-12

**Authors:** Berengère Benoit, Pierre Letourneau, Vincent Verdier, Angélique Viney, Cécile Barnel, Karim Chikh, Oriane Vitalis, Audrey Jalabert, Sandra Wagner, Manolo Laiola, Natalia Alencar-De-Pinho, Florence Thirion, Stéphanie Chanon, Aurélie Vieille-Marchiset, Sarah Teixeira, Fabien Subtil, Sylvie Bin, Alice Beau, Claudie Pinteur, Ziad A. Massy, Pamela Dugues, Jean-Claude Alvarez, Amine Larabi, Griet Glorieux, Filipe De Vadder, Christophe Soulage, François Leulier, Hubert Vidal, Laetitia Koppe

**Affiliations:** a Université Lyon1, INSERM, INRAE, CarMeN, Pierre Bénite, France; b Department of Nephrology and Nutrition, Hospices Civils de Lyon, Hôpital Lyon-Sud, Pierre-Bénite, France; c Departement of Nephrology, University Hospital of Reims, Reims, France; d Laboratoire de Biochimie, Hospices Civils de Lyon, Hôpital Lyon-Sud, Pierre-Bénite, France; e Université de Lorraine, INSERM CIC 1433, Nancy CHRU, Inserm U1116, FCRIN INI-CRCT, Nancy, France; f Université Paris-Saclay, INRAE, MGP, Jouy-en-Josas, France; g Centre for Research in Epidemiology and Population Health (CESP), Paris-Saclay University, Versailles Saint-Quentin University, INSERM UMRS 1018, Clinical Epidemiology Team, Villejuif, France; h Service de Biostatistique, Hospices Civils de Lyon, Pierre-Bénite, France; i Service Recherche et Épidémiologie Cliniques, Pôle de Santé Publique, Hospices Civils de Lyon, France; j Association de l’Utilisation des Reins Artificiels (AURA), Paris, and Division of Nephrology, Ambroise Paré University Hospital, AP-HP, Boulogne-Billancourt/Paris, France; k Department of Pharmacology and Toxicology, Raymond Poincaré Hospital, AP-HP, Garches; Centre for Research in Epidemiology and Population Health (CESP), Team MOODS, Inserm UMRS 1018, UVSQ-Paris-Saclay University, Montigny-le-Bretonneux, France; l Nephrology Unit, Department of Internal Medicine and Pediatrics, Ghent University Hospital, Ghent, Belgium; m Institute of Functional Genomics of Lyon, ENS Lyon, CNRS, Université Claude Bernard Lyon 1, UMR5242, Lyon, France

**Keywords:** Probiotics, chronic kidney disease, uremic toxins, gut microbiota, low-protein diet

## Abstract

Low-protein diets (LPD) are recommended in chronic kidney disease (CKD) to reduce disease progression. However, their clinical efficacy and safety are debated due to the risk of protein-energy wasting. A deeper mechanistic understanding is therefore required. Herein, the metabolic effects of LPD in both murine models and a randomized controlled trial in nondiabetic CKD patients were investigated, focusing on glucose homeostasis, plasmatic uremic toxin (UTs) levels, gut microbiota remodeling, and endocrine adaptations. In both experimental and clinical settings, LPD improved glucose tolerance and significantly decreased circulating levels of gut-derived UTs while reducing body weight (−33% weight gain in mice and a decrease in body mass index of ~−0.5 kg/m^2^ in humans). These metabolic improvements were associated with alterations in gut microbiota composition and function, including the downregulation of microbial pathways involved in aromatic amino acid biosynthesis. In both mice and patients, LPD triggered a significant hepatic induction of fibroblast growth factor 21 (FGF21), an endocrine regulator of amino acid deficiency (+2.9-fold in human and 28-fold in mice) FGF21 levels correlated negatively with lean mass and positively with fat mass and glycemic control, supporting a dual role in metabolic adaptation and catabolic signaling. To mitigate the adverse nutritional effects of LPD, we administered *Lactiplantibacillus plantarum* ^WJL^ (Lp^WJL^), a probiotic previously found to enhance growth of under nutritional stress in CKD mice. Lp^WJL^ restored circulating amino acid levels, suppressed FGF21 induction (−26%) and stress-related biosynthetic responses, and preserved body weight (+247% weight gain) and composition, without impairing the benefits of LPD on kidney and metabolic parameters. The present findings identify UTs and FGF21 as crucial factors of the metabolic response to LPD, and support microbiota-targeted strategies, such as Lp^WJL^ supplementation, to enhance LPD efficacy. Clinical trials are, however, required to confirm their relevance in CKD management.

## Introduction

Chronic kidney disease (CKD) is projected to become the fifth leading cause of death by 2040, underscoring the urgency of early interventions to slow its progression.^1^ When kidney function declines, the clearance of metabolites is impaired, leading to the accumulation of uremic toxins (UTs), many of which are derived from gut microbial metabolism.^2-5^ These UTs contribute to CKD complications, notably by altering glucose metabolism and adipose tissue function.^6-9^ While new pharmacological therapies such as sodium-glucose cotransporter-2 (SGLT2) inhibitors, mineralocorticoid receptor antagonists (MRAs), and glucagon-like peptide-1 (GLP-1) receptor agonists have considerably improved kidney survival, residual risks remain. In this context, nutritional strategies, and particularly protein restriction, is a cornerstone in CKD management.^10^ ^,^ ^11^ The Kidney Disease Improving Global Outcomes (KDIGO) and Kidney Disease Outcomes Quality Initiative (KDOQI) guidelines recommend low-protein diets (LPD) and very LPD (VLPD) supplemented with ketoanalogues (KA); their clinical efficacy is, however, debated due to poor adherence and potential risks of protein-energy wasting (PEW).^11^ ^,^ ^12^ A deeper mechanistic understanding is therefore critical to enhance benefits of LPD on the metabolic and kidney function while minimizing adverse outcomes.

Under healthy conditions, LPD has been shown to influence glucose homeostasis;^13-17^ however, its metabolic effects in CKD are poorly characterized.^18-21^ A key hypothesis is that LPD may exert metabolic benefits in CKD by reducing circulating UTs, including urea and microbial metabolites such as tyrosine-derived and tryptophan-derived uremic toxins (e.g., *p*-cresyl sulfate and indoxyl sulfate, respectively).^3^ ^,^ ^22^ ^,^ ^23^ LPD may reduce UT production by lowering dietary precursors (e.g., aromatic amino acids) and modulating gut microbiota composition. Of note, a plant-based LPD appeared to decrease the abundance of UT precursor-producing bacterial species.^2^ Small interventional clinical studies^24^ ^,^ ^25^ have also suggested that microbiota remodeling under LPD may further suppress UT generation, potentially linking protein intake to systemic glucose homeostasis regulation. LPD has also been found to modulate fibroblast growth factor 21 (FGF21) expression, a hepatic hormone that responds to protein restriction by promoting food intake, increasing energy expenditure, and enhancing lipid oxidation. FGF21 upregulation may function as an adaptive metabolic signal to maintain energy balance during nutrient scarcity.^15^ ^,^ ^16^ ^,^ ^26^ However, chronically elevated FGF21 levels have been associated with adverse outcomes in CKD^27^ and with PEW-related conditions.^28^ ^,^ ^29^

Emerging data suggest that gut microbiota modulation may influence FGF21 responses to protein restriction, highlighting a new axis of nutrient sensing with therapeutic implications.^26^ Probiotic interventions have been reported to modulate gut microbiota composition, restoring metabolic and nutritional balance.^30^ ^,^ ^31^ In a previous study, we demonstrated that the probiotic strain *Lactiplantibacillus plantarum WJL (*Lp^WJL^) (formerly named *Lactobacillus plantarum WJL*) enhances growth hormone (GH) sensitivity and increases insulin growth factor-1 (IGF-1) levels in protein-restricted juvenile mice.^32^ ^,^ ^33^ Identified for its growth-promoting effects in Drosophila, Lp^WJL^ mimics the benefits of a complex microbiota and mitigates stunting under malnutrition.^32^ ^,^ ^33^ However, its efficacy in CKD and its interaction with FGF21 signaling remain unexplored.

Therefore, the objectives of the present study were: 1) to investigate the metabolic effects of LPD in CKD, by assessing UT levels, glucose homeostasis, gut microbiota composition, and FGF21 response using murine models and a randomized controlled trial in non-diabetic patients; and 2) to evaluate whether Lp^WJL^ supplementation could mitigate the potential LPD-induced metabolic stress and PEW in murine models.

## Methods

*See Supplementary Methods for full details.*

### Animal experiments

All animal procedures were approved by an institutional review board (#2021112217245161 and #2022080515563596) and complied with ARRIVE guidelines. LPD impact (6.1% protein) was evaluated in two murine models of CKD: male C57BL/6J undergoing adenine-induced nephropathy (0.25% adenine-enriched A04 diet; SAFE, Augy, France) and 5/6 nephrectomy (5/6 Nx). In the latter, Lp^WJL^ supplementation (3.10^8^ CFU in 20% maltodextrin + 2% dextrose) or vehicle was administered by oral gavage. Details regarding animal procedures, sample collection, body composition analysis, metabolic studies, UTs quantification, and histology are provided in the Supplementary Methods.

### Metabolomic analyses

Metabolomic profiling was performed at the METANUTRIBIOTA platform (Lyon, France). Serum and liver samples were analyzed using the MxP® Quant 500 kit (Biocrates Life Sciences AG, Innsbruck, Austria) on a Xevo TQ-XS triple quadrupole mass spectrometer coupled with an Acquity ultra-performance liquid chromatography (UPLC) system (Waters, Milford, MA, USA). This targeted platform quantifies up to 630 metabolites across 26 biochemical classes using LC-mass spectrometry/mass spectrometry (MS/MS) and flow injection analysis-MS/MS. Sample preparation and analytical procedures for both serum and liver metabolomics are detailed in the Supplementary Methods.

### Human study

The KETO-GUT study was an open-label, randomized 1:1, controlled clinical trial designed to assess the effects of a VLPD supplemented with KA (referred to as the LPD group) compared to a normal diet (ND) on plasmatic UT levels, gut microbiota composition, and metabolic outcomes in patients with advanced nondiabetic CKD. All participants provided written informed consent. The protocol was approved by an institutional review board (CPP Ouest II-Angers, RCB 2019-02225-30) and registered on ClinicalTrials.gov (NCT03959228). Full methodological details are available in the Supplementary Methods.

Participants were recruited from the Nephrology Department at the *Hôpital Lyon Sud* (Hospices Civils de Lyon, France). The inclusion criteria were: age 18–80 y, stage 4–5 CKD (estimated glomerular filtration rate [eGFR] < 30 mL/min/1.73 m^2^), body mass index (BMI) between 18 and 33 kg/m^2^, and absence of diabetes. The exclusion criteria included dialysis, kidney transplantation, pregnancy, malnutrition (serum albumin < 38 g/L), prior colorectal surgery, advanced colorectal cancer, diagnosed inflammatory gut disease, and recent (within 1 month) use of antibiotics, probiotics, or prebiotics.

All participants received individualized dietary counseling in line with contemporary CKD nutrition guidelines.^12^ During a 3-month run-in phase, dietary protein intake was standardized to 0.8 g/kg/d. Participants were then randomized to either continue this diet (ND group) or switch to an isocaloric LPD (0.4 g/kg/d) supplemented with KA (1 tablet/5 kg/d) for 3 months. Protein intake was assessed monthly via 24-hour urinary nitrogen excretion and calculated using the Maroni formula.^34^ In addition, detailed dietary intake was assessed every 3 months using a 3-d food recall, with nutrient content analyzed using the French CIQUAL food composition database.

Biological samples (blood, urine, feces) were collected at randomization (T0) and after the intervention (T3). Fecal samples were processed according to SOP 5 of the International Human Microbiome Standards. Routine blood biochemistry was performed, and UT levels were quantified by ultra-performance LC (UPLC).^2^

### DNA extraction, 16S rRNA sequencing, and microbiota analysis

Genomic DNA was extracted from human fecal and mice cecal samples using validated protocols.^2^ ^,^ ^35^ The V3–V4 region of the 16S rRNA gene was amplified and sequenced on an Illumina MiSeq platform (Illumina, San Diego, CA, USA). Sequence processing, taxonomic assignment, and diversity analyses were performed using standard bioinformatic pipelines. Functional metagenomic predictions were generated using the Phylogenetic Investigation of Communities by Reconstruction of Unobserved States 2 (PICRUSt2) tool in human.^36^

### Statistical analysis

All statistical analyses were performed using R (https://www.r-project.org) and GraphPad Prism 10 (https://www.graphpad.com). Statistical methods are described in detail in the Supplementary Methods. A *p*-value < 0.05 was considered statistically significant.

## Results

### Impact of LPD on glucose homeostasis and uremic toxicity in CKD mice

**Figure 1.**
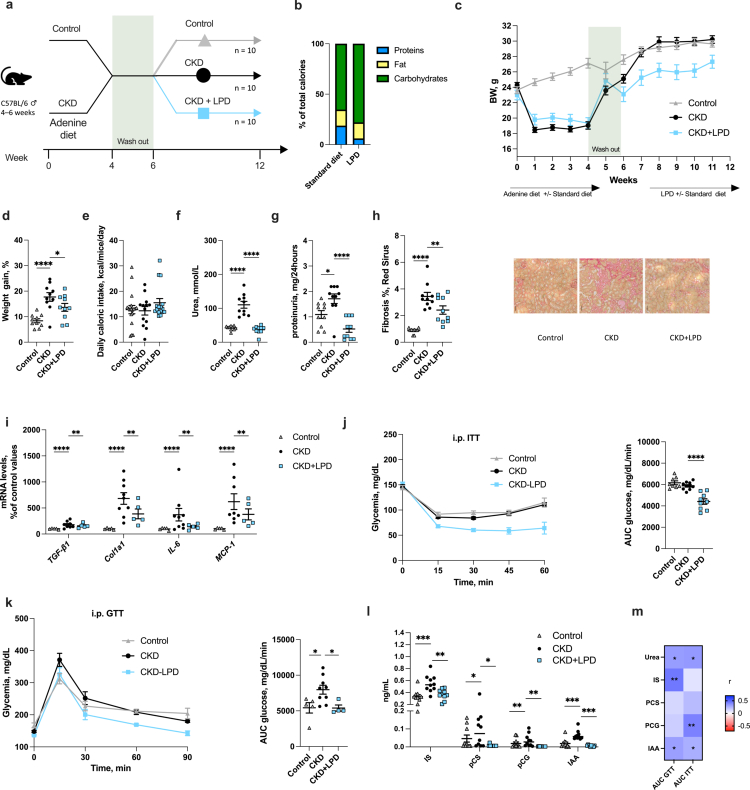
Low-protein diet reduces uremic burden and improves metabolic and renal outcomes in CKD mice. (a) Experimental design: CKD was induced by a 4-week adenine-enriched diet followed by a 2-week washout period, after which mice were randomized to receive either a standard protein diet (18.8% protein) or a low-protein diet (LPD; 6.1% protein) for 6 weeks (*n* = 10 per group). (b) Macronutrient composition of the diets expressed as a percentage of total caloric intake. (c) Body weight evolution during the intervention (*n* = 10 per group). (d) Total body weight gain at endpoint (*n* = 10 per group). (e) Daily energy intake per mouse (*n* = 10 per group). (f) Plasma urea concentration (*n* = 10 per group). (g) Urinary protein-to-creatinine ratio (*n* = 10 per group). (h) Quantification of renal fibrosis by Sirius red staining and representative micrographs (scale bar, 100 μm) (*n* = 10 per group). (i) Relative renal mRNA expression of transforming growth factor beta 1 (Tgfb1), collagen type I alpha 1 (Col1a1), interleukin-6 (Il6), and monocyte chemoattractant protein-1 (Mcp1), normalized to TATA-box binding protein (Tbp) and expressed relative to sham mice (*n* = 5 for controls, *n* = 8–9 for CKD, *n* = 5 for CKD + LPD). (j) Blood glucose levels during intraperitoneal insulin tolerance test (ITT, 0.5 U/kg) (*n* = 8 for controls, *n* = 10 for CKD, *n* = 9 for CKD + LPD) and (k) glucose tolerance test (GTT, 2 g/kg) (*n* = 5 for controls, *n* = 9 for CKD, *n* = 4 for CKD + LPD), with corresponding area under the curve (AUCs). (l) Plasma concentrations of microbiota-derived uremic toxins: indoxyl sulfate (IS), *p*-cresyl sulfate (PCS), *p*-cresyl glucuronide (PCG), and indole-3-acetic acid (IAA). (*n* = 10 per group). (m) Spearman's correlation matrix between GTT and ITT AUCs; color represents Spearman's correlation coefficient (r). Data are presented as mean ± SEM. Statistical analysis was performed using one-way ANOVA followed by Bonferroni post hoc test. *p < 0.05, **p < 0.01, ***p < 0.001; ****p < 0.0001.

To evaluate the therapeutic potential of a LPD in CKD, its effects on glucose homeostasis, kidney function, and circulating UT levels were assessed in a murine model of CKD. CKD was induced in mice through administration of an adenine-enriched diet, followed by six weeks of isocaloric feeding with identical fat content, using either a standard-protein diet (18.8% protein) or a low-protein diet (6.1% protein), in which corn starch served as the non-nitrogenous energy substitute. (Table S1, Figure 1a–b). Baseline body weights (BW) were similar across the 3 groups (Figure 1c). Mice with CKD maintained on a standard diet progressively regained BW during the follow-up period and reached levels similar to controls by week 12. In contrast, CKD + LPD exhibited persistently lower BW despite a trend toward increased food intake compared to CKD mice on a standard diet (Figure 1c–e). Body composition analysis revealed a significant reduction in lean mass, liver weight, and kidney weight in CKD + LPD compared to their standard diet counterparts (Table 1).

**Table 1.** Body composition in CKD mice induced by adenine diet under standard or low protein diets.

	Sham (*n* = 10)			CKD (*n* = 10)			CKD-LPD (*n* = 10)		
eWAT (% total BW)	1.7 ± 0.1***			1.2 ± 0.1			2.3 ± 0.1***		
Gastrocnemius (% total BW)	0.5 ± 0.0			0.5 ± 0.0			0.5 ± 0.0		
Liver (% total BW)	4.3 ± 0.1*			4.7 ± 0.1			3.8 ± 0.1***		
Kidney (% total BW)	1.2 ± 0.0***			0.8 ± 0.0			0.6 ± 0.1***		
Heart (% total BW)	0.5 ± 0.0*			0.6 ± 0.0			0.6 ± 0.0		
Lean mass, g	23.1 ± 0.8			24.7 ± 0.4			19.9 ± 0.7***		
Fat mass, g	3.8 ± 0.4*			2.7 ± 0.3			3.3 ± 0.2		

Lean mass and fat masses were measured at endpoint using time-domain NMR (MiniSpec, Bruker). eWAT: epididymal white adipose tissue. Data are expressed as mean ± SEM. Statistical significance was assessed using one-way ANOVA followed by Bonferroni post hoc test: *p < 0.05; ***p < 0.001 vs CKD.

Notably, CKD + LPD mice exhibited significant improvements in kidney parameters, including reduced plasma urea levels, lower proteinuria, and attenuated kidney fibrosis, along with a decreased expression of pro-inflammatory and pro-fibrotic genes compared to CKD (Figure 1f–i). In addition, the metabolic profile was improved in CKD + LPD mice regarding glucose regulation, as evidenced by improved intraperitoneal insulin tolerance test (i.p. ITT) and glucose tolerance test (i.p. GTT) compared to CKD mice fed on a standard diet (Figure 1j–k). Circulating levels of representative UTs were significantly decreased in CKD + LPD mice, reaching values similar to those of controls (Figure 1l). Furthermore, plasma UT levels positively correlated with the area under the curve (AUC) of the i.p. GTT and ITT AUC (Figure 1m). These data indicate that LPD mitigates the systemic burden of UTs, improves kidney outcomes as well as glucose metabolism in the context of CKD in mice.

### The impact of LPD on glucose homeostasis and uremic toxicity in patients with CKD

**Figure 2.**
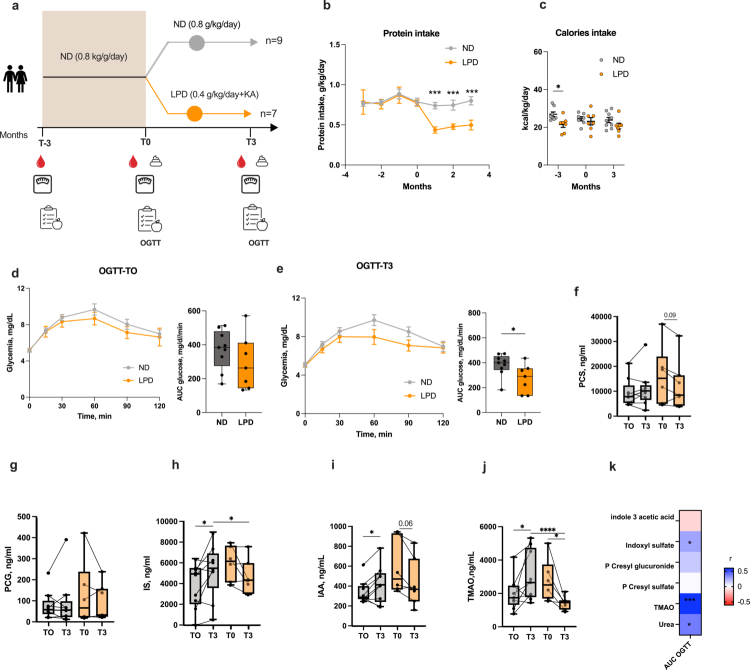
Short-term low-protein diet reduces uremic toxin accumulation and improves glucose tolerance in CKD patients. (a) Study design. After a 3-month run-in period, non-diabetic patients with CKD stage 4-5 were randomized to a normal-protein diet (ND, 0.8 g/kg/d, *n* = 9) or a very low-protein diet supplemented with ketoanalogues (LPD, 0.4 g/kg/d, *n* = 7) for 3 months. (b) Protein intake estimated by 24-hour urinary urea excretion and (c) caloric intake estimated by 3-d dietary recall. (d) Blood glucose levels during an oral glucose tolerance test (OGTT, 75 g of glucose) and corresponding area under the curve (AUC) at baseline and after 3 months. (e–j) Boxplots (with median) showing plasma concentrations of uremic toxins: (f) *p*-cresyl sulfate (PCS), (g) *p*-cresyl glucuronide (PCG), (h) indoxyl sulfate (IS), (i) indole-3-acetic acid (IAA), and (j) trimethylamine *N*-oxide (TMAO) at baseline (T0) and after 3 months (T3). (*n* = 9 for ND and *n* = 6 for LDP) (k) Correlation matrix showing associations between OGTT-AUC and circulating uremic toxins; color intensity represents Spearman's correlation coefficients (r). Data are presented as mean ± SEM or as boxplots with median. Statistical significance was assessed using two-tailed paired or unpaired *t*-tests, or Wilcoxon/Mann–Whitney *U* tests depending on data distribution. Normality was evaluated using the Shapiro–Wilk test. *p < 0.05, **p < 0.01, ***p < 0.001, ****p < 0.0001.

To assess the translational relevance of our preclinical findings, we conducted a randomized controlled trial, the KETO-GUT study, designed to evaluate the impact of a LPD, without caloric restriction, on plasmatic UT levels and metabolic outcomes in non-diabetic patients with CKD. In accordance with clinical guidelines,^11^ the LPD was supplemented with KA to ensure nutritional adequacy. Following a 3-month run-in period during which all participants received a normal protein intake (ND; 0.8 g/kg/d), 18 patients with stage 4–5 CKD were randomized to either an isocaloric LPD (0.4 g/kg/d + KA, 1 tablet per 5 kg BW) or to continue on the ND for an additional 3-month period (Figure 2a, Fig. S1). After randomization, 2 patients were excluded due to withdrawal of consent, and 16 patients were finally analyzed, with baseline characteristics presented in Table S2.

Body composition, clinical parameters, and metabolic profiles were assessed from fasting blood samples, and an oral GTT (OGTT) was performed at T0 and T3 (Figure 2a). As expected, protein intake estimated by 24 h urine and by 3-d dietary recall differed significantly between groups, while caloric intake remained stable despite a trend toward a decrease in the LPD group (Figure 2b–c and Table 2). Although the intervention duration was limited, eGFR decline was more pronounced in the ND group (Table 2). Importantly, patients receiving the LPD exhibited a significant reduction in serum urea (−3 mmol/L), along with a decrease in plasma parathyroid hormone and an increase in serum bicarbonate (Table 2, Table S3).

**Table 2.** Changes in body composition, renal parameters, and metabolic markers in CKD patients enrolled in the KETO-GUT study and receiving either a standard diet (ND, 0.8 g/kg/d of protein) or a low-protein diet (LPD; 0.4 g/kg/d of protein supplemented with ketoacid analogues).

	ND T0	*n*	LPD T0	*n*	*p*-value	ND T3	*n*	LPD T3	*n*	*p*-value	D ND TO-T3	*p*-value	D LPD TO-T3	*p*-value
Body composition														
Body weight (kg)	74.0 ± 9.9	9	74.4 ± 11.1	7	0.94	74.1 ± 9.3	9	73.0 ± 10.6	7	0.83	0.1 ± 0.5	0.83	−1.4 ± 0.5	0.02
Body mass index (BMI kg/m^2^)	25.3 ± 2.9	9	26.7 ± 4.0	7	0.45	25.4 ± 2.6	9	26.2 ± 4.1	7	0.66	0.1 ± 0.2	0.59	−0.5 ± 0.1	0.02
Fat mass (kg)	22.0 ± 5.1	9	25.6 ± 10.97	7	0.45	21.1 ± 4.3	9	25.3 ± 10.0	6	0.36	−0.9 ± 0.7	0.23	0.3 ± 1.0	0.79
Fat mass (%)	30.1 ± 7.5	9	33.8 ± 13.1	7	0.52	29.2 ± 7.6	9	35.2 ± 12.1	6	0.31	−0.9 ± 0.8	0.30	1.3 ± 1.41	0.39
Lean tissue index (kg)	42.6 ± 10.7	9	38.6 ± 11.3	7	0.48	44.4 ± 12.4	9	35.9 ± 10.3	6	0.17	1.8 ± 1.0	0.11	−1.7 ± 1.5	0.32
Lean mass (%)	57.3 ± 10.0	9	52.9 ± 17.97	7	0.57	59.4 ± 10.7	9	51.2 ± 15.5	6	0.29	2.0 ± 1.2	0.14	−1.9 ± 2.2	0.42
Handgrip strength (kg)	32.8 ± 9.4	9	30.5 ± 15.33	7	0.74	32.6 ± 9.5	9	29.8 ± 13.5	7	0.65	−0.2 ± 0.7	0.76	−0.7 ± 0.8	0.40
Diet (3-d dietary recall)														
Dietary fiber intake (g/d)	18.2 ± 2.9	9	18.9 ± 6.1	7	0.81	17.3 ± 1.5	9	23.3 ± 4.7	7	0.03	−0.9 ± 1.7	0.62	4.4 ± 1.9	0.06
Protein intake (g/kg/d)	0.79 ± 0.14	9	0.77 ± 0.11	7	0.40	0.80 ± 0.15	9	0.50 ± 0.16	7	<0.01	0.0 ± 0.2	0.44	−0.3 ± 0.2	< 0.01
Diet (by 24 h urine)														
Sodium intake (g/d)	8.0 (5.4–14.4)	9	4.5 (2.4–10.2)	7	<0.001	7.1 (5.2–13.4)	9	4.1 (2.5–7.1)	7	<0.001	−1.0 ± 0.6	0.13	−0.5 ± 1.1	0.38
Renal parameters														
eGFR (ml/min/1.73 m^2^)	21.4 ± 2.3	9	22.4 ± 4.8	7	0.74	19.6 ± 7.3	9	21.7 ± 4.6	7	0.49	−1.9 ± 0.8	0.05	−0.7 ± 1.0	0.49
Serum urea (mmol/L)	15.4 ± 3.9	9	13.3 ± 2.3	7	0.21	16.8 ± 4.4	9	10.1 ± 3.4	6	<0.01	1.4 ± 0.6	0.06	−3.0 ± 1.0	0.03
Glucose Control														
Hemoglobin A1c (%)	5.5 (5.2–6.1)	8	5.5 (5.2–6.0)	7	0.80	5.6 (5.2–6.3)	9	5.4 (5.2–5.6)	7	0.39	0.0 ± 0.1	0.88	−0.2 ± 0.1	0.03
Plasma glucose basal (mmol/L)	5.1 ± 0.5	9	5.2 ± 0.5	7	0.79	5.1 ± 0.7	9	5.0 ± 0.4	7	0.81	−0.1 ± 0.1	0.51	−0.2 ± 0.2	0.28
Plasma glucose 2 h (mmol/L)	6.9 ± 1.3	9	6.6 ± 2.6	7	0.75	7.0 ± 1.6	9	6.8 ± 0.5	7	0.85	−0.0 ± 0.7	0.99	0.2 ± 0.7	0.78
Plasma insulin basal (μU/mL)	9.5 ± 6.3	9	10.1 ± 6.9	7	0.87	11.6 ± 4.9	9	10.0 ± 4.5	7	0.51	2.1 ± 1.0	0.03	−0.1 ± 1.4	0.96
Plasma insulin 2 h (μU/mL)	75 (7–162)	9	36 (26–103)	7	0.30	49 (17–109)	9	57 (17–177)	7	0.99	−17.7 ± 13.3	0.20	14.1 ± 16.5	0.47
AUC glucose 120 min (mmol·min/L)	371 ± 117	9	293 ± 160	7	0.31	382 ± 29	9	273 ± 114	7	0.05	11.6 ± 27.8	0.69	−20.3 ± 53.9	0.72
AUC insulin 120 min (μU·min/mL)	7150 ± 2137	9	5766 ± 3025	7	0.33	6147 ± 1974	9	6964 ± 3301	7	0.58	−1003 ± 924	0.31	1198 ± 1752	0.52
Insulin Sensitivity														
Matsuda Index	3.8 (1.6–6.8)	9	4.1 (2.6–20.2)	7	0.60	3.8 (1.8–4.8)	9	3.8 (2.2–6.4)	7	0.54	−0.6 ± 0.4	0.44	−2.3 ± 2.0	0.30
HOMA-IR	1.4 (1.0–6.2)	9	2.8 (0.0–5.3)	7	0.85	2.4 (1.4–6.5)	9	2.4 (0.5–3.2)	7	0.99	0.5 ± 0.2	0.04	−0.2 ± 0.4	1.00
Disposition Index (IGI × ISI)	2.5 (1.0–8.0)	9	2.9 (0.1–13.8)	7	0.99	2.6 (1.5–5.6)	9	3.0 (0.3–7.6)	7	0.54	−0.51 ± 0.5	0.50	−1.2 ± 1.8	0.81
Beta-cell function														
HOMA-beta	102 (54–237)	9	107 (3–314)	7	0.92	151 (67.−322)	9	134 (28–360)	7	0.76	47 ± 16	0.03	33 ± 19	0.16
Insulinogenic Index (*β*-cell)	0.9 ± 0.4	9	0.7 ± 0.4	7	0.39	0.8 ± 0.3	9	0.9 ± 0.5	7	0.93	0.0 ± 0.1	0.91	0.2 ± 0.2	0.41
FGF21 control														
FGF21 basal (pg/mL)	62 (0–946)	9	130 (0–308)	7	0.76	50 (0–688)	9	474 (0–773)	7	0.09	−90 ± 62	0.31	293 ± 105	0.05
FGF21 2 h (pg/mL)	84 (0–1145)	9	149 (11–336)	7	0.99	82 (11–685)	9	531 (24–1023)	7	0.02	−169 ± 101	0.07	449 ± 128	0.02
FGF21 4 h (pg/mL)	117 (0–1774)	9	342 (15–744)	7	0.30	205 (10–746)	9	958 (31–1469)	7	0.02	−91 ± 161	0.65	541 ± 168	0.02
AUC FGF21 240 min (pg·min/mL)	13,769 (4680–167,630)	9	18,469 (4888–36,982)	7	0.61	16772 (3695–154,184)	9	64,545 (7947–99,352)	7	0.09	3982.0 ± 23416	0.91	40,332 ± 10312	0.02

Values are expressed as mean ± standard deviation (SD) or as median (min–max) for variables with non-normal distribution. n: number of patients. Within-group comparisons between baseline and follow-up were performed using two-tailed paired t-tests or Wilcoxon signed-rank tests, as appropriate. Between-group comparisons were conducted using two-tailed unpaired t-tests or Mann–Whitney *U* tests, depending on the distribution of the data. Normality was assessed using the Shapiro–Wilk test.

Participants in the LPD group displayed similar profiles to those observed in the CKD mice model, including significant reductions in BW (~−1.4 kg) and BMI (~−0.5 kg/m^2^; Table 2) with a nonsignificant trend toward lower caloric intake despite nutritional support (20.7 ± 1.5 vs 23.8 ± 1.4 kcal/kg/d; *p* = 0.14).(Figure 2c) At T0, glucose homeostasis was similar between groups; however, LPD feeding improved glucose tolerance without modification of insulin sensitivity (HOMA-IR) or *β*-cell function (HOMA-*β*), as compared to the ND group (Figure 2d–e, Table 2). Consistent with preclinical data, LPD intervention led to a significant reduction in specific circulating gut-derived UTs. Plasma levels of aromatic amino acids, including tryptophan, tyrosine, and phenylalanine, remained unchanged (Figure 2f–i, Table S3). Notably, plasmatic UT levels correlated with glycemic parameters, supporting the hypothesis that LPD improves glucose metabolism in CKD, at least in part, through modulation of the UT-related burden (Figure 2k).

### LPD reshapes gut microbiota composition in mice and patients with CKD

**Figure 3.**
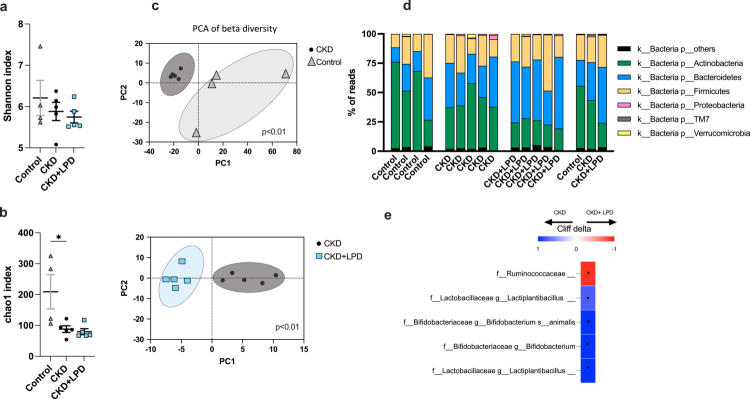
Low-protein diet modulates gut microbiota diversity and composition in CKD mice. (a) Shannon index and (b) Chao1 index across Sham, CKD, and CKD + LPD groups. Statistical analysis was performed using one-way ANOVA followed by Bonferroni post hoc test. Data are presented as mean ± SEM (*n* = 4 for controls, *n* = 5 for CKD, *n* = 5 for CKD + LPD). (c) Principal coordinates analysis (PCoA) based on Bray–Curtis dissimilarity (*β*-diversity) comparing CKD vs. Sham (top) and CKD vs. CKD + LPD (bottom). Ellipses represent the 95% confidence intervals of sample distribution within each group. Axes indicate the percentage of variance explained by the first two principal components. Statistical significance was assessed using PERMANOVA (*n* = 4 for controls, *n* = 5 for CKD, *n* = 5 for CKD + LPD). (d) Relative abundance of bacterial phyla in fecal samples from individual mice (*n* = 4 for controls, *n* = 5 for CKD, *n* = 5 for CKD + LPD). (e) Heatmap scaled to Cliff's delta effect size at the species level, highlighting taxa identified by 16S rRNA sequencing as significantly enriched in CKD or CKD + LPD (*p* < 0.05, Cliff's delta > 0.1). Taxonomic annotations: p_ for phylum, g_ for genus, and f_ for family. False discovery rates (q-values) were calculated using the Mann–Whitney *U* test with Benjamini–Hochberg correction. Circles indicate q < 0.1 and *p < 0.05 (*n* = 5 for CKD, *n* = 5 for CKD + LPD). Blue indicates enrichment in CKD, red indicates enrichment in CKD + LPD.

To investigate whether the reduction in gut-derived UT levels observed under LPD was influenced by gut microbiota remodeling rather than solely by decreased availability of metabolic precursors, we conducted 16S rRNA gene sequencing of fecal samples from both the CKD mice and human. In mice at the end of the diet, Chao1 index is higher in control mice compare to CKD mice but *α*-diversity metrics (Shannon and Chao1 indices) did not differ significantly between CKD and CKD + LPD (Figure 3a–b), yet *β*-diversity analysis revealed a significant shift of microbiota composition (Figure 3c). Taxonomic profiling at the phylum level confirmed marked compositional shifts (Figure 3d), as CKD + LPD mice exhibited significantly reduced relative abundances of genera belonging to *Bifidobacterium animalis*, *Bifidobacterium*, and *Lactiplantibacillus,* together with an increased relative abundance of *Ruminococcaceae.* (Figure 3e, Table S4).

**Figure 4.**
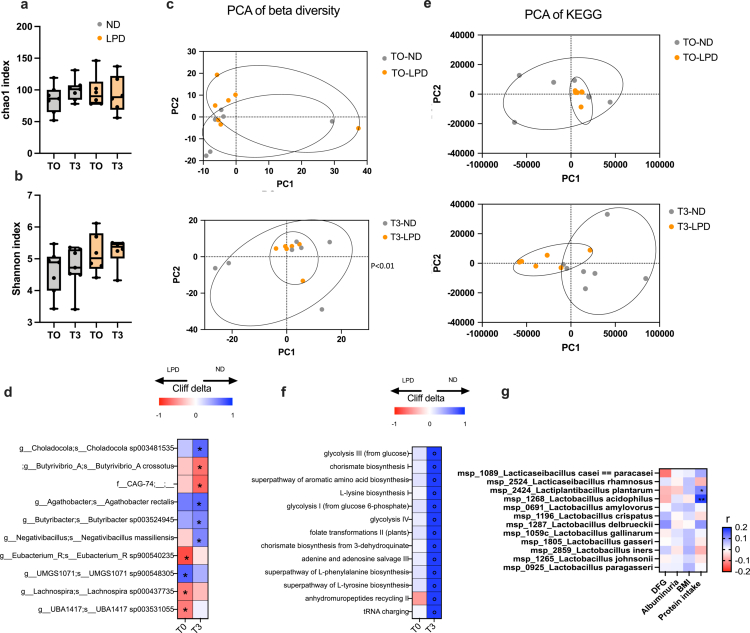
Short-term low-protein diet modifies gut microbiota structure and function in CKD patients. (a) Chao1 index and (b) Shannon index at baseline (T0) and after 3 months (T3) in CKD patients receiving a normal-protein diet (ND, *n* = 7) or a low-protein diet (LPD, *n* = 6). Data are presented as as boxplots with median. Statistical significance was assessed using paired or unpaired t-tests, or Wilcoxon/Mann–Whitney *U* tests depending on data distribution. (c) Principal coordinates analysis (PCoA) based on Bray–Curtis dissimilarity (*β*-diversity) at baseline (T0, top) and after 3 months (T3, bottom). Ellipses indicate the spatial distribution of samples within each group. Axes represent the percentage of variance explained by the first two principal components. Statistical significance was assessed using PERMANOVA. (d) Heatmap scaled to Cliff's delta effect size at the species level, showing taxa identified by 16S rRNA sequencing as significantly enriched in ND or LPD at T0 and T3 (*p* < 0.05, Cliff's delta > 0.1). Taxonomic annotations: p_ for phylum, g_ for genus, f_ for family. Blue indicates enrichment in ND, red indicates enrichment in LPD. Asterisks indicate statistically significant differences between groups (*p < 0.05) (e) Principal coordinates analysis (PCoA) based on KEGG pathway-level dissimilarities at T0 (top) and T3 (bottom), as predicted by PICRUSt2. Ellipses represent the spatial distribution of samples within each group. Statistical significance was assessed using PERMANOVA. (f) Heatmap scaled to Cliff's delta effect size showing the top 13 bacterial enzymes predicted by PICRUSt2 as significantly associated with ND or LPD at T0 and T3 (*p* < 0.05, Cliff's delta > 0.1). False discovery rates (q-values) were estimated using the Mann–Whitney test with Benjamini–Hochberg correction. Circles indicate q < 0.1. Blue indicates enrichment in ND, red indicates enrichment in LPD. (g) Spearman's correlation matrix (r) between fecal *Lactiplantibacillus* abundance and clinical parameters (protein intake, BMI, DGL, and albuminuria) in the CKD-REIN cohort^2^. *p < 0.05, **p < 0.01.

Similarly, in patients with CKD, LPD induced substantial and significant modifications in gut microbial composition. While no major difference was observed regarding *α*- or *β*-diversity (Figure 4a–c), the taxonomic profile was significantly modified. Notably, a significant increase in the abundance of *Choladocola, Butyrivibrio, Agathobacter, Negativibacillus,* and of an unidentified genus was observed in the LPD group compared with the ND group (Figure 4d, Table S5).

To obtain insight into the functional implications of these taxonomic shifts, we used PICRUSt2^36^ to infer metagenomic content from 16S ribosomal RNA gene profiles. This approach predicted the abundance of microbial genes and assigned enzyme classification numbers, allowing for pathway-level functional annotation based on the Kyoto Encyclopedia of Genes and Genomes (KEGG) ontology. We then performed principal component analysis (PCA) on the resulting pathway abundance matrix. This analysis revealed a clear difference between the LPD and ND groups, indicating substantial remodeling of microbial metabolic capacity (Figure 4e, Table S6). The super pathway of aromatic amino acid biosynthesis was found to be the most downregulated pathway in the LPD group among others (Figure 4f). Altogether, these findings demonstrated that LPD extensively reshapes the gut microbiota in CKD, potentially contributing to reduced UTs production.

### *Lactiplantibacillus plantarum* ^WJL^ supplementation limits nutritional deterioration in CKD mice under a low-protein diet

Based on previous evidence that Lp^WJL^ promotes growth under malnutrition, and on our observation of *Lactiplantibacillus* depletion in CKD + LPD mice,^32^ ^,^ ^33^ we reanalyzed shotgun metagenomic data from the CKD-REIN cohort^2^ to examine the correlation between dietary protein intake and *Lactiplantibacillus* abundance. *L. plantarum* and *L. acidophilus* levels were positively correlated with protein intake (Figure 4g), suggesting that this species may be sensitive to protein availability. These findings support the hypothesis that microbiota-targeted interventions, such as Lp^WJL^ supplementation, could alleviate LPD-related nutritional risks in CKD.

**Figure 5.**
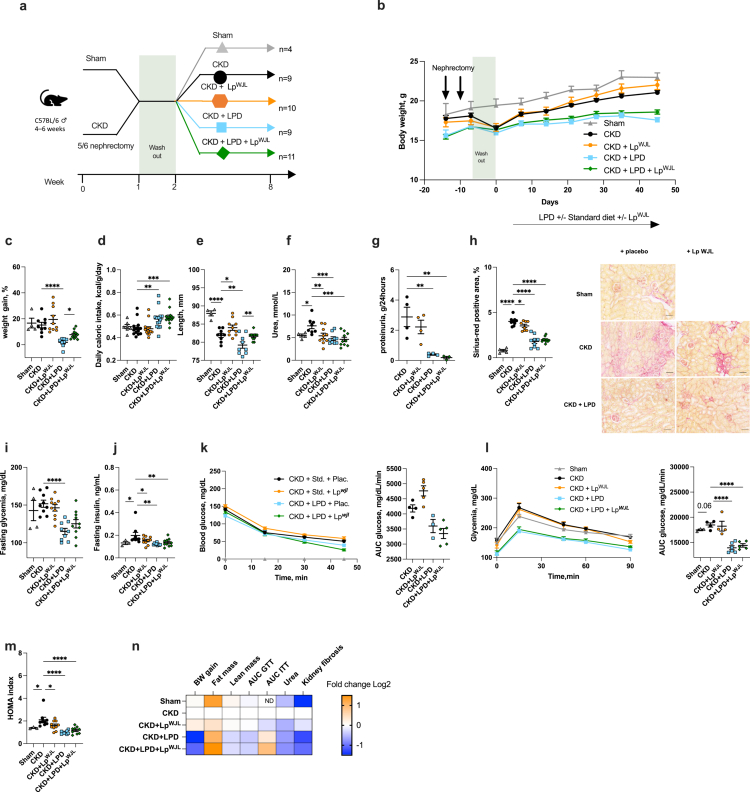
*Lactiplantibacillus plantarum* WJL supplementation limits body weight loss and improves metabolic parameters in CKD mice under low-protein diet. (a) Experimental design: CKD was induced by a two-step 5/6 nephrectomy followed by a 1-week washout period, after which mice were randomized to receive either a standard-protein diet (18.8% protein) or a low-protein diet (LPD; 6.1% protein) for 6 weeks, with or without supplementation with *Lactiplantibacillus plantarum* WJL (Lp^WJL^; 3.10^8^ CFU, 5 d per week). (*n* = 4 for Sham, *n* = 9 for CKD, *n* = 10 for CKD + LP^WJL^; *n* = 9 for CKD + LPD, *n* = 11 for CKD + LPD + Lp^WJL^) (b) Body weight evolution during the intervention. (*n* = 4 for Sham, *n* = 9 for CKD, *n* = 10 for CKD + LP^WJL^, *n* = 9 for CKD + LPD, *n* = 11 for CKD + LPD + Lp^WJL^). (c) Total body weight gain at endpoint (*n* = 4 for Sham, *n* = 9 for CKD, *n* = 10 for CKD + LP^WJL^, *n* = 9 for CKD + LPD, *n* = 11 for CKD + LPD + Lp^WJL^). (d) Daily energy intake per gram of body weight per mouse (*n* = 4 for Sham, *n* = 9 for CKD, *n* = 10 for CKD + LP^WJL^, *n* = 9 for CKD + LPD, *n* = 11 for CKD + LPD + Lp^WJL^). (e) Length at endpoint. (*n* = 4 for Sham, *n* = 9 for CKD, *n* = 10 for CKD + LP^WJL^; *n* = 9 for CKD + LPD, *n* = 11 for CKD + LPD + Lp^WJL^). (f) Plasma urea concentration. (*n* = 4 for Sham, *n* = 8 for CKD, *n* = 10 for CKD + LP^WJL^; *n* = 9 for CKD + LPD, *n* = 10 for CKD + LPD + Lp^WJL^). (g) 24 h-urinary proteinuria (*n* = 4 for CKD, *n* = 4 for CKD + LP^WJL^, *n* = 4 for CKD + LPD, *n* = 3 for CKD + LPD + Lp^WJL^). (h) Quantification of renal fibrosis by Sirius red staining and representative micrographs (scale bar, 100 μm) (*n* = 4 for Sham, *n* = 9 for CKD, *n* = 8 for CKD + LP^WJL^, *n* = 9 for CKD + LPD, *n* = 11 for CKD + LPD + Lp^WJL^). (i) Fasting blood glucose (*n* = 4 for Sham, *n* = 9 for CKD, *n* = 10 for CKD + LP^WJL^, *n* = 9 for CKD + LPD, *n* = 10 for CKD + LPD + Lp^WJL^), and (j) fasting insulin levels (*n* = 3 for Sham, *n* = 9 for CKD, *n* = 10 for CKD + LP^WJL^, *n* = 9 for CKD + LPD, *n* = 10 for CKD + LPD + Lp^WJL^). (k) Blood glucose levels during intraperitoneal insulin tolerance test (ITT, 0.5 U/kg), with corresponding area under the curve (AUC) (*n* = 4 for CKD, *n* = 5 for CKD + LP^WJL^; *n* = 3 for CKD + LPD, *n* = 5 for CKD + LPD + Lp^WJL^). (l) Blood glucose levels during glucose tolerance test (GTT, 2 g/kg), with corresponding area under the curve (AUC) (*n* = 3 for Sham, *n* = 4 for CKD, *n* = 4 for CKD + LP^WJL^, *n* = 6 for CKD + LPD, *n* = 5 for CKD + LPD + Lp^WJL^). (m) Homeostasis model assessment of insulin resistance (HOMA-IR) (*n* = 3 for Sham, *n* = 8 for CKD, *n* = 10 for CKD + LP^WJL^, *n* = 9 for CKD + LPD, *n* = 9 for CKD + LPD + Lp^WJL^). (n) Log₂ fold change in parameters related to body composition, glucose metabolism, and kidney function. Data are presented as mean ± SEM. Statistical analysis was performed using one-way ANOVA followed by Bonferroni post hoc test. *p < 0.05, **p < 0.01, ***p < 0.001, ****p < 0.0001.

To test this, 5/6 Nx CKD mice were administered oral Lp^WJL^ (3.10^8^ CFU) or vehicle, five times per week for six weeks, under either a standard diet or LPD (Figure 5a, Table S1). Consistent with the adenine-induced model, LPD feeding in 5/6 Nx mice resulted in significant weight loss and signs of nutritional impairments, despite increased caloric intake. Lp^WJL^ supplementation effectively limited weight loss and improved growth, and increased fat mass, without any change in energy intake (Figure 5b–e; Table 3). Under a standard diet, Lp^WJL^ also reduced plasma urea levels and attenuated kidney fibrosis (Figure 5f–h), although no additional protective effect on kidneys was observed under LPD conditions, likely because LPD had already largely normalized kidney parameters. Notably, despite changes in body composition, Lp^WJL^ did not impair the beneficial metabolic effects of LPD (Figure 5k–m).

**Table 3.** Body composition in CKD mice induced by 5/6 nephrectomy, under control or low-protein diet, with or without supplementation with Lactiplantibacillus plantarum WJL.

	Sham (*n* = 4)			CKD (*n* = 9)			CKD + LP^WJL^ (*n* = 10)			CKD + LPD (*n* = 9)			CKD + LPD + LPWJL (*n* = 11)		
eWAT (% total BW)	1.24 ± 0.09			1.19 ± 0.07*			1.23 ± 0.09			1.5 ± 0.05££			1.72 ± 0.09$		
Gastrocnemius muscle (% total BW)	1.16 ± 0.02			1.18 ± 0.02			1.2 ± 0.02			1.14 ± 0.02			1.2 ± 0.05		
Liver (% total BW)	4.47 ± 0.15			3.38 ± 0.43***			3.76 ± 0.05			2.96 ± 0.06£££			3.08 ± 0.07		
Kidneys (% total BW)	1.28 ± 0.05			0.76 ± 0.03***			0.7 ± 0.03			0.62 ± 0.03££			0.74 ± 0.04$		
Heart (% total BW)	0.48 ± 0.00			0.51 ± 0.01*			0.52 ± 0.02			0.53 ± 0.01			0.53 ± 0.01		
Lean mass, g	18.9 ± 0.4			17.5 ± 0.1*			18.1 ± 0.2			14.5 ± 0.1£££			14.7 ± 0.1		
Fat mass, g	1.53 ± 0.08			0.61 ± 0.02***			0.89 ± 0.04€			1.28 ± 0.02£££			1.73 ± 0.02$$$		

eWAT: epididymal white adipose tissue; sc WAT: Subcutaneous white adipose tissue. Data are expressed as mean ± SEM. Statistical significance was assessed using one-way ANOVA followed by Bonferroni post hoc test: *p < 0.05; ***p < 0.001 sham vs CKD; $*p *< 0.05, $$$*p *< 0.001: CKD + LPD vs CKD + LPD + LP^WJL^; ££*p *< 0.01; £££*p *< 0.001 CKD vs CKD + LPD ; €*p *< 0.05 CKD vs CKD + LP^WJL^.

We previously demonstrated that in malnourished juvenile mice without CKD, Lp^WJL^ restored growth and nutritional status by activating the growth hormone (GH)/insulin-like growth factor-1 (IGF-1) axis.^32^ ^,^ ^33^ However, in CKD mice, LPD reduced GH/IGF-1 signaling, and Lp^WJL^ administration did not significantly modify circulating IGF-1 levels or hepatic GH receptor expression (Fig. S2a-b), suggesting that the observed benefits may be mediated through GH/IGF-1–independent mechanisms.

Overall, these findings support a functional synergy between LPD and Lp^WJL^ supplementation in CKD, whereby Lp^WJL^ alleviated LPD-induced nutritional impairment while preserving, and possibly enhancing, kidney function and metabolic benefits of dietary protein restriction (Figure 5n).

### *Lactiplantibacillus plantarum* ^WJL^ mitigates the FGF21 metabolic stress response elicited by low-protein diet in CKD mice

**Figure 6.**
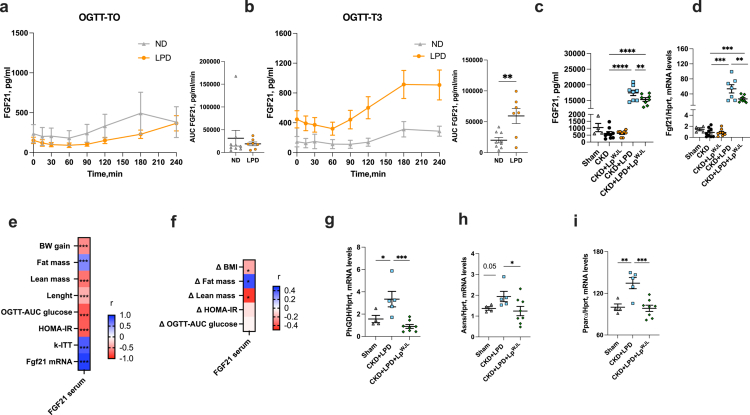
*Lactiplantibacillus plantarum* WJL supplementation modulates circulating and hepatic FGF21 expression in CKD mice under low-protein diet. (a) Plasma concentration of fibroblast growth factor 21 (FGF21) in CKD patients receiving a normal-protein diet (ND, *n* = 9) or a low-protein diet (LPD, *n* = 7) at baseline (T0) and (b) after 3 months (T3). Data are presented as mean ± SEM. Statistical significance was assessed using two-tailed unpaired t-tests (c) Plasma FGF21 concentration in CKD mice under LPD with or without *Lactiplantibacillus plantarum* WJL (Lp^WJL^) supplementation (*n* = 4 for Sham, *n* = 9 for CKD, *n* = 8 for CKD + LP^WJL^, *n* = 8 for CKD + LPD, *n* = 8 for CKD + LPD + Lp^WJL^). (d) Relative hepatic mRNA expression of *Fgf21*, normalized to TATA-box binding protein (*Tbp*) and expressed relative to sham mice in CKD mice under LPD with or without Lp^WJL^ supplementation (*n* = 4 for Sham, *n* = 9 for CKD, *n* = 8 for CKD + LP^WJL^, *n* = 7 for CKD + LPD, *n* = 8 for CKD + LPD + Lp^WJL^). Data are presented as mean ± SEM. Statistical analysis was performed using one-way ANOVA followed by Bonferroni post hoc test. (e, f) Spearman's correlation matrices between baseline plasma FGF21 levels and body composition or glucose-related parameters in mice (e) and in humans (f); color intensity reflects Spearman's correlation coefficient (r). (g–i) Relative hepatic mRNA expression of (g) *Phgdh* (phosphoglycerate dehydrogenase), (h) *Asns* (asparagine synthetase), and (i) *PPARα (*peroxisome proliferator-activated receptor alpha), normalized to *Tbp* and expressed relative to sham controls (*n* = 4 for Sham, *n* = 5 for CKD + LPD, *n* = 8 for CKD + LPD + Lp^WJL^). Data are presented as mean ± SEM. Statistical analysis was performed using one-way ANOVA followed by Bonferroni post hoc test. *p < 0.05, **p < 0.01, ***p < 0.001, ****p < 0.0001.

To better understand the beneficial effects of Lp^WJL^ supplementation in the context of CKD, and given the established role of FGF21 as a biomarker of the metabolic stress response to low-protein intake, we examined this pathway in both patients with CKD and mouse models. In humans, plasma FGF21 levels remained stable under ND, but increased significantly at T3 after the LPD (+291% from T0, Figure 6a–b, Table 2). During OGTT, FGF21 levels were significantly higher in LPD-treated CKD patients compared to those maintained on a ND. Similarly, in 5/6 Nx mice, LPD feeding led to increased circulating FGF21 levels and upregulation of hepatic *Fgf21* expression (Figure 6c–d). In both humans and mice, baseline FGF21 levels were negatively correlated with height and lean mass, while elevated FGF21 was correlated with improved glucose homeostasis (Figure 6e–f), consistent with previous reports that FGF21 enhances insulin sensitivity but contributes to muscle wasting in the absence of caloric restriction. Strikingly, Lp^WJL^ supplementation alleviated the LPD-induced hepatic FGF21 response in CKD mice (Figure 6c–d), an effect that was accompanied by improved nutritional status.

We explored whether compensatory mechanisms such as endogenous amino acid biosynthesis were activated as a response to protein restriction in CKD. We specifically examined hepatic expression of key genes involved in the synthesis of non-essential amino acids, including *Asns* (asparagine synthetase) and *3pgd* (3-phosphoglycerate dehydrogenase), which contribute to the production of asparagine and serine, respectively. Both genes were significantly upregulated in CKD mice under LPD, reflecting a hepatic nutrient stress–driven metabolic adaptation. Remarkably, Lp^WJL^ supplementation significantly reduced the expression of *Asns* and *3pgd* in CKD mice, restoring their levels to those observed in Sham mice (Figure 6g–h). We also measured the nutrient-responsive transcription factor peroxisome proliferator-activated receptor alpha (*PPARα*), which was attenuated by Lp^WJL^ under LPD conditions (Figure 6i). Both pathways converge on the induction of FGF21.

Altogether, these findings suggested that Lp^WJL^ mitigates the hepatic nutrient stress in CKD associated with protein restriction, thereby supporting better nutritional status, potentially through mechanisms involving both attenuation of FGF21 signaling and reduced reliance on endogenous amino acid biosynthesis.

### *Lactiplantibacillus plantarum* ^WJL^ supplementation alters liver and plasma metabolite profiles under a low-protein diet

**Figure 7.**
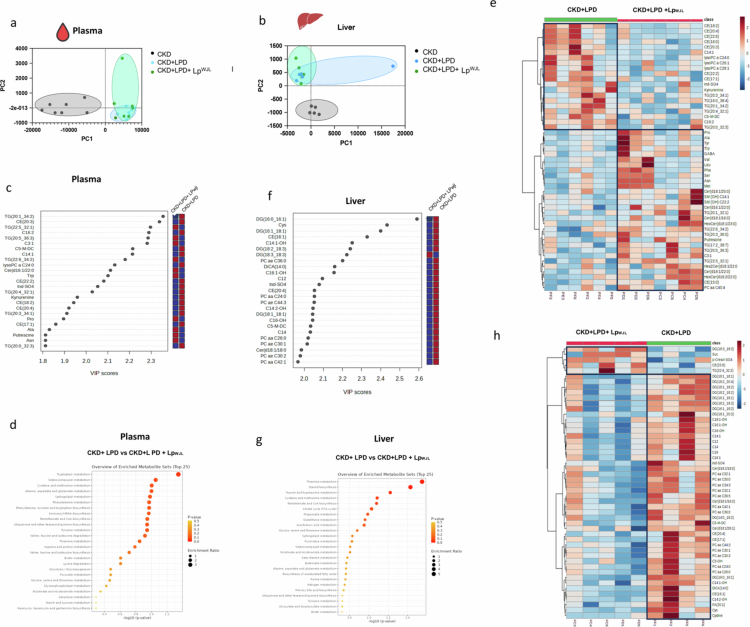
*Lactiplantibacillus plantarum* WJL supplementation combined with low-protein diet alters blood and liver metabolomes in CKD mice. (a, b) Principal component analysis (PCA) of metabolites in (a) plasma (*n* = 7 for CKD, *n* = 7 for CKD + LPD, *n* = 7 for CKD + LPD + Lp^WJL^) and (b) liver samples (*n* = 5 for CKD, *n* = 5 for CKD + LPD, *n* = 5 for CKD + LPD + Lp^WJL^) from CKD mice receiving a low-protein diet (LPD), with or without *Lactiplantibacillus plantarum* WJL (Lp^WJL^) supplementation. Ellipses indicate the spatial distribution of samples within each group. Axes represent the percentage of variance explained by the first two principal components. Statistical significance was assessed using PERMANOVA. (c, f) Variable importance in projection (VIP) plots showing the top 25 metabolites (VIP > 1.8) discriminating groups in (c) plasma and (f) liver, based on partial least squares discriminant analysis (PLS-DA) using MetaboAnalyst comparing CKD-LPD vs. CKD-LPD + Lp^WJL^ groups. (d, g) Metabolite set enrichment analysis (MSEA) of identified metabolites mapped to KEGG human metabolic pathways in (d) plasma and (g) liver, comparing CKD-LPD vs. CKD-LPD + Lp^WJL^ groups. (e, h) Heatmaps showing relative abundance of detected metabolites in (e) plasma and (h) liver samples. Color scale represents normalized intensity values ranging from blue (low abundance) to red (high abundance) comparing CKD-LPD vs. CKD-LPD + Lp^WJL^ group.

Based on prior evidence that Lp^WJL^ enhances gut peptidase expression and amino acid absorption in drosophila,^37^ we hypothesized that Lp^WJL^ alleviates nutrient stress in CKD, increasing amino acid availability, particularly under conditions of protein restriction. To test this, we performed semi-targeted metabolomic profiling of plasma and liver samples from CKD mice (Tables S7–S8). PCA revealed distinct metabolic clustering primarily driven by diet, with a lesser influence from probiotic administration (Figure 7a–b). Partial least squares discriminant analysis (PLS-DA) identified discriminant metabolites based on variable importance in projection (VIP) scores (Fig. S3a, d), indicating that LPD profoundly reshapes hepatic and systemic metabolism, particularly in amino acid–related pathways (Fig. S3b–c, e–f).

In plasma, amino acid concentrations remained stable or increased in CKD mice under LPD (Table S7). This paradoxical stability of circulating amino acids, even under protein restriction, has already been reported in other LPD challenges^38^ and likely reflects compensatory mechanisms at the tissue level, particularly in muscle and liver.^39^ ^,^ ^40^ Indeed, in CKD mice under LPD, several non-essential amino acids, including glutamine, were elevated in the liver compare to CKD mice, consistent with nitrogen recycling and *de novo* synthesis (Fig. S3 d–f, Table S8). These findings suggest that activation of hepatic nutrient stress pathways may (e.g., FGF21) represent more relevant biomarkers of metabolic adaptation than circulating amino acid concentrations. Nevertheless, Lp^WJL^ supplementation under LPD in CKD mice increased circulating amino acid levels, reduced hepatic amino-acids levels, and significantly reduced tryptophan-derived UTs, including indoxyl sulfate and kynurenine (Figure 7c–e). To refine interpretation, we applied MetaboINDICATOR™, which aggregates metabolite data into functional ratios and composite indicators. Among 232 computed indicators, the top 15 VIP-ranked features (Fig. S4a) consistently indicated downregulation of tryptophan metabolism in Lp^WJL^-supplemented CKD mice under LPD. A similar, albeit less pronounced, reduction in hepatic tryptophan metabolism was also observed (Figure 7f–h; Fig. S4b).

Importantly, the increase in plasma amino acids by Lp^WJL^ (Fig. S4c) occurred independently of changes in intestinal barrier or peptide transport, as expression levels of *occludin* and the *peptide transporter PEPT1* (*SLC15A1*) remained unchanged (Fig. S5a–b). However, LPD combined with Lp^WJL^ increased ileal crypt proliferation, consistent with prior reports of microbiota-mediated epithelial renewal (Fig. S5c) suggesting a specific trophic effect, as previously demonstrated.^33^

As expected, Lp^WJL^ supplementation did not significantly alter the overall gut microbiota structure, but selectively increased the abundance of *Lactiplantibacillus* species, mirroring changes observed in CKD mice under LPD (Fig. S5d–e, Table S9). To assess whether these functional effects could be attributed to the metabolic potential of Lp^WJL^ itself, we reanalyzed previously published whole-genome sequencing data.^41^ Genes involved in amino acid metabolism and derivatives accounted for ~7% of the Lp^WJL^ genome (Fig. S5f). Notably, Lp^WJL^ harbors complete biosynthetic pathways for several essential amino acids, including tryptophan, methionine, and lysine (Fig. S5g), supporting its contribution to the elevated levels of circulated amino acid found in CKD mice under LPD. Interestingly, genomic reconstruction indicated that the indole pathway in Lp^WJL^ was incomplete, suggesting that the strain lacks the capacity to produce indole.

Taken together, these findings indicated that Lp^WJL^ supplementation under protein restriction increases amino acid levels and reduces circulating of tryptophan-derived UTs, thereby supporting a better nutritional status without compromising the kidney and metabolic benefits of dietary protein restriction in CKD (Fig. S5i).

## Discussion

The present translational study, combining an in-depth analysis in CKD patients with complementary *in vivo* models found that LPD improved metabolic parameters and reduced UT production by reshaping the composition and function of gut microbiota. However, LPD also induced nutritional stress, as highlighted by an increased hepatic FGF21 response and elevated plasma FGF21 levels. Notably, targeted modulation of the microbiota via Lp^WJL^ supplementation mitigated FGF21-mediated stress with increasing circulating amino acids and thereby limiting weight loss. LPD combined with Lp^WJL^ thus emerges as a promising approach to enhance both adherence and metabolic tolerance in CKD.

The role of LPDs in CKD management and in the general population remains under active investigation and is currently debated.^42^ While a deleterious impact of lean mass has previously been reported, some studies, including the present preclinical CKD model, found a protective effect on kidney function and an extended lifespan, which is often not achieved in patients with severe CKD due to uremia-induced anorexia. Herein, despite nutritional support, we observed a trend toward reduced energy intake that remained below recommended levels (25–35 kcal/kg body weight per day^11^), and weight loss, indicating inadequate compensation. These findings highlight the need for complementary strategies beyond caloric supplementation to limit the potential adverse effects of LPD and strengthen their clinical relevance in CKD.

Disorders of glucose homeostasis affect nearly half of patients with CKD, even in the absence of diabetes.^43^ Previous studies^18^ ^,^ ^19^ suggested that LPD may mitigate this impairment, a finding confirmed herein. Notably, the observed metabolic improvement was associated with reduced circulating levels of urea and UTs. Key UTs, such as urea and *p*-cresyl sulfate, have been found to be related to insulin resistance and *β*-cell dysfunction in multiple *in vitro* and *in vivo* models.^6^ ^,^ ^7^ ^,^ ^43^ A large-scale study by Xie et al. (*n* = 1.3 million US veterans) also reported that elevated serum urea correlates with a higher risk of incident diabetes.^44^ While the present findings support this correlation, they remain observational. Other factors, such as altered phosphate/calcium metabolism, acid–base imbalance, medications, inactivity, or diet, may also contribute. Herein, LPD was associated with decreased PTH and increased plasma bicarbonate, which could influence glucose metabolism. The co-administration of KA may also influence kidney function, gut microbiota, and UT levels, although these effects were not specifically investigated in this study. KA are converted into their corresponding amino acids through transamination, mainly in the liver but also in peripheral tissues such as muscle. This process reutilizes excess circulating nitrogen to form amino acids, thereby reducing urea production and potentially limiting UT precursors. Thus, KA supplementation primarily acts at the level of hepatic metabolism rather than through a direct intestinal effect. We cannot completely exclude a direct contributory role of KA on UTs levels, as suggested by small preliminary cohorts,^45^ nor can we rule out a potential direct effect on gut microbiota composition through specific molecular components. Moreover, modulation of nitrogen metabolism may indirectly affect intestinal microbiota balance and permeability.^46^ ^,^ ^47^ Future studies directly comparing LPD alone versus LPD plus KA in controlled experimental models would be valuable to disentangle these mechanisms. However, the similarity of results between humans (with supplementated with KAion) and mice (without KA supplementation) suggests that this role is not crucial. Finally, in the present study LPD did not only reduce UT precursors but also reshaped gut microbial function, particularly by downregulating amino acid biosynthetic pathways. These results support the hypothesis that LPD improve metabolism in CKD, in part by limiting UT production through microbiota modulation, a finding consistent with prior clinical data showing that protein intake in CKD positively correlates with UT-producing microbial taxa.^2^ We did not observe significant change in circulating levels of plasma aromatic amino acid in CKD patients under an LPD, which may reflect host compensatory mechanisms aimed at maintaining amino acid homeostasis. In addition, we did not explore the full metabolic fate of aromatic amino acids using integrated metabolomic approaches. While the gut origin of *p*-cresyl sulfate and indoxyl sulfate is well established, other UTs derived from the kynurenine pathway are produced both by the host and the gut microbiota.^48^ This dual origin requires further investigation and may explain the limited variation of these metabolites in response to dietary interventions in patients with CKD. Our clinical study only included 16 patients, and this small sample size may explain why differences in the concentrations of some UTs were not statistically significant, given the large interindividual variability in their plasma levels. Additional studies with larger cohorts combining metagenomics and metabolomics will be necessary to confirm these observations and better characterize these metabolic pathways.

Several studies have demonstrated that gut microbiota composition and function are altered in CKD.^2^ ^,^ ^5^ However, most available data are derived from patients with end-stage kidney disease,^49^ ^,^ ^50^ whereas fewer less studies have investigated gut microbiota alterations in non-dialysis CKD populations.^2^ ^,^ ^5^ Recent studies,^2^ ^,^ ^5^ including ours,^2^ suggest that the CKD gut microbiota is enriched in bacteria carrying genes involved in UT production. The key role of the gut microbiota has been supported by *in vitro* studies, showing that fecal samples from CKD patients produce higher levels of *p*-cresol.^35^ Indole production depends on substrate availability, such as tryptophan or nitrite.^35^ ^,^ ^51^ Moreover, UTs such as indoxyl sulfate may stimulate the production of nitrite by enterocytes, which in turn promotes the growth of specific bacteria, including *Escherichia coli* strains carrying tryptophanase, thereby increasing indole production and potentially creating a vicious cycle that contributes to CKD progression.^51^ The potential of probiotics for modulating UTs, particularly aromatic amino acid–derived metabolites, to reduce disease progression and metabolic disturbances has been highlighted in recent reviews.^3^ ^,^ ^35^ ^,^ ^52-57^ Mechanistically, several pathways have been identified. For instance, *Lactobacillus* species such as *L. johnsonii* have been shown to attenuate kidney injury by modulating the aryl hydrocarbon receptor (AhR) pathway through increased production of indole-3-aldehyde.^58^ Similarly, *Bacteroides ovatus* has been reported to reduce renal fibrosis by promoting hyodeoxycholic acid production via *Clostridium scindens*, leading to activation of G protein-coupled bile acid receptor 1 (TGR5), inhibition of the farnesoid X receptor, and increased glucagon-like peptide-1 (GLP-1) secretion, ultimately conferring renal protection.^59^ In addition, several probiotics, including *L* *actobacillus johnsonii, Lactobacillus murinus, Lactobacillus vaginalis, Lactobacillus reuteri,* and *Bifidobacterium animalis*, may influence renal function through tryptophan-derived metabolites that interact with host receptors modulate glomerular integrity.^60^ However, very few interventions have demonstrated clear clinical efficacy or improvements on hard clinical outcomes, largely due to limited and short-duration studies. In addition, only a small number of studies have explored the links between gut microbiota, nutritional status, and PEW, mainly in dialysis populations.^61^ Therefore, further investigations are needed to better understand these interactions across all stages of CKD.

Consistent with findings in undernutrition models,^32^ ^,^ ^33^ herein, Lp^WJL^ improved nutritional status in CKD mice. As opposed to previous studies, significant involvement of the GH/IGF-1 axis was not observe herein, possibly due to the use of older animals with CKD, in which this pathway is known to be downregulated, resistant and less pivotal than during the growth period.^62^ The contribution of downstream effectors such as JAK/STAT5 remains to be investigated. Our hypothesis focuses on the ability of Lp^WJL^ to modulate the host's amino acid metabolism under LPD. However, its mechanisms of action remain incompletely understood and probably several mechanisms are involved, including the ability of Lp^WJL^ to directly produce amino acids, a reduction in hepatic nutrient sensing, and a specific trophic effect of Lp^WJL^. Originally isolated from *Drosophila*, Lp^WJL^ promotes larval growth during nutrient deprivation, likely via mTOR activation, which is sensitive to branched amino acid. Inactivation of the amino acid transporter *slimfast* abolishes this effect,^63^ and Lp^WJL^ has been found to enhance dietary protein assimilation by inducing intestinal peptidases.^37^ Some studies have demonstrated the capacity of *Lactobacillus* species, including the WJL strain, to produce several essential amino acids, although this has not been specifically investigated under LPD conditions.^64^ We, however, did not measure portal amino acid concentrations in CKD mice under LPD with Lp^WJL^ and therefore cannot confirm a direct intestinal origin of the increased circulating amino acids. Intestinal permeability does not appear to be a major contributor, as we failed to observed significant changes; however, this was not assessed using dedicated functional tests. Moreover, genetic inference^41^ and *in vitro* data^65^ supports the hypothesis that Lp^WJL^ increases the systemic availability of essential amino acids, particularly tryptophan, one of the predominant amino acids in both Lp^WJL^-fed larvae and CKD mice receiving Lp^WJL^-supplemented LPD. FGF21 as a critical physiological checkpoint coordinating growth in response to nutritional stress^38^ and we observed that Lp^WJL^ attenuate hepatic nutritional stress by affecting both ATF4-dependent signaling and PPARα pathways that drive FGF21 activation under LPD. Previous studies have reported that restriction of only one or a few specific amino acid, especially tryptophan, was sufficient to trigger systemic metabolic responses, akin to those observed with overall protein restriction, including FGF21 induction, which is a key endocrine sensor of amino acid imbalance.^15^ Notably, FGF21 activation requires the microbiota: germ-free mice fail to present a FGF21 response to protein restriction.^26^ Another hypothesis in that, Lp^WJL^ may act through immune–epithelial crosstalk. Its cell wall components activate NOD2 in intestinal epithelial cells, a mechanism shown to support growth during undernutrition.^33^ Cell wall–derived signals from Lp^WJL^, including muramyl dipeptide and mifamurtide, increase epithelial proliferation via NOD2, an intracellular receptor that senses peptidoglycan. This effect is restricted to the intestinal epithelium, as it is lost in gut-specific NOD2 knockout mice but preserved in hepatocyte-specific knockouts.^33^ Although not directly investigated here, our observation of epithelial remodeling with increased proliferation (Fig. S5c) supports a similar trophic effect,^33^ which will require further dedicated studies. This could also explain the beneficial effects of Lp^WJL^ on growth without major modification of the gut microbiota and its lack of detection in mice treated with Lp^WJL^. Finally, the mechanisms by which Lp^WJL^ reduces tryptophan-derived UTs remain unclear. This may involve several mechanisms that profoundly modify amino acid metabolism, including reduced bacterial catabolism of aromatic amino acids, improved intestinal trophicity, and potential modulation of host hepatic metabolism, such as sulfation, which was out of the scope of the present study.

The increase in FGF21 and its role in CKD remains unclear.^15^ ^,^ ^26^ ^,^ ^66^ While moderate increases may be beneficial in metabolic diseases such as diabetes and metabolic dysfunction-associated steatotic liver disease,^67^ sustained elevation, as seen in CKD and under LPD, may be harmful. High FGF21 levels are associated with CKD progression,^68^ yet some studies suggest a compensatory role. For example, FGF21 knock-out worsens LPD-induced kidney damage through inflammatory pathways.^69^ Also, pharmacological administration of FGF21 analogs has shown renoprotective effects in models of diabetic nephropathy.^70^ In the present study, elevated FGF21 correlated with improved metabolic parameters but also with weight loss, raising questions regarding its dual effects and therapeutic potential in CKD. The physiological role of FGF21 remains complex and context-dependent,^67^ and this study does not fully resolve whether its elevation is protective or maladaptive. We therefore interpret FGF21 primarily as a biomarker of nutritional stress rather than a causal factor. Further mechanistic studies are required. In particular, studies using knockout models should be conducted, including hepatocyte-specific Fgf21 knockout mice. These approaches will help determine whether the renoprotective and/or body weight–reducing effects observed in CKD mice under a LPD are lost in the absence of FGF21. They will also allow assessment of whether treatment with FGF21 analogs reproduces nephroprotective and/or body weight–reducing effects in this model. Moreover, the metabolic effects of LPD are likely not solely explained by the amino acid–FGF21 axis. Other pathways, including microbiota-derived metabolites (potentially influenced by Lp^WJL^), bile acid–Farnesoid X Receptor (FXR) signaling,^26^ ^,^ ^71^ and additional metabolic networks that remain to be identified, may also contribute and need to be explored to fully decipher the mechanisms underlying these responses.

This study has limitations. First, experiments were conducted in male mice, limiting generalizability of the findings without further validation; given the known influence of sex on the response to both LPD and Lp^WJL^ species.^72^ Reducing dietary protein necessarily increases carbohydrate intake, which could modify several metabolic pathways. However, the metabolic profile, including glucose tolerance, is improved under this diet, suggesting a limited adverse effect. Moreover, our previous data showed that macronutrient-specific feed efficiency indicate that metabolic adaptations under LPD are not explained by improved carbohydrate utilization, but instead reflect selective, protein-centered adaptations.^38^ ^,^ ^72^ Thus, the observed metabolic effects primarily arise from protein restriction rather than increased carbohydrate intake. Second, regarding the modulation of protein intake and aromatic amino-acid pathways, PICRUSt2 predictions are informative but have important limitations, as does 16S rRNA sequencing, and these findings should be confirmed by shotgun metagenomics. Third, the absence of *Lactobacillus* reduction in CKD patients under LPD, in contrast to mice, may reflect anatomical sampling differences, as *Lactobacillus* preferentially localize to the ileum,^73^ whereas only distal fecal samples were analyzed in humans, in contrast to cecal samples in mice. However, in a metagenomic cohort including 240 patients with CKD, which allows species-level resolution, *L. plantarum* abundance positively correlated with protein intake, supporting the biological plausibility of a decrease under LPD. Finally, we did not perform longitudinal analyses in mice or humans and therefore cannot determine whether the observed microbial and metabolic effects could be sustained over time. We hypothesize that LPD combined with Lp^WJL^ may exert synergistic long-term effects, maintaining nephroprotection while mitigating metabolic stress signals, particularly in vulnerable populations such as the elderly; however, this requires confirmation in dedicated long-term studies.

## Conclusion

The present findings identify UTs and FGF21 as crucial factors of the metabolic response to LPD, and support microbiota-targeted strategies, such as Lp^WJL^ supplementation, to enhance LPD efficacy and limit PEW. Clinical trials are, however, required to confirm their relevance in CKD management.

## Supplementary Material

Supplementary Material Fig_S1.pdf

Supplementary Material Fig_S2.pdf

Supplementary Material Fig_S3.pdf

Supplementary Material Fig_S4.pdf

Supplementary Material Fig_S5.pdf

Supplementary Material ARRIVEchecklistVf.docx

Supplementary Material Supplementary_Figure.docx

Supplementary Material Supplementary clean version .docx

Supplementary Material TableFinalversion_For reviewer.xlsx

## Data Availability

The data supporting the findings of this study are available from the corresponding author upon reasonable request. 16S rRNA sequencing datasets have been deposited in the French repository “Recherche.data.gouv.fr” and are accessible at the following link: https://doi.org/10.57745/EWUIIW.

## Patient and public involvement

Patients and public were not involved in the design or conduct or reporting or dissemination of the present study.

## Resource availability

**Lead Contact**

The data described in this manuscript will be made available upon reasonable request to Pr. Laetitia Koppe (laetitia.koppe@chu-lyon.fr), contingent upon appropriate ethics approval, approval of the research proposal, and execution of a Data Transfer Agreement (DTA).

## Materials availability

This study did not generate new unique reagents.
