# Supplementary figures and images for: Lactiplantibacillus plantarum WJL ameliorates chronic kidney disease by inhibiting fibroblast growth factor 21 adaptive stress response via low protein diet
Source: Gut Microbes. 2026 Jul 12;18(1):2696622. doi: 10.1080/19490976.2026.2696622 (PMC13367091; doi:10.1080/19490976.2026.2696622)

Figure S1

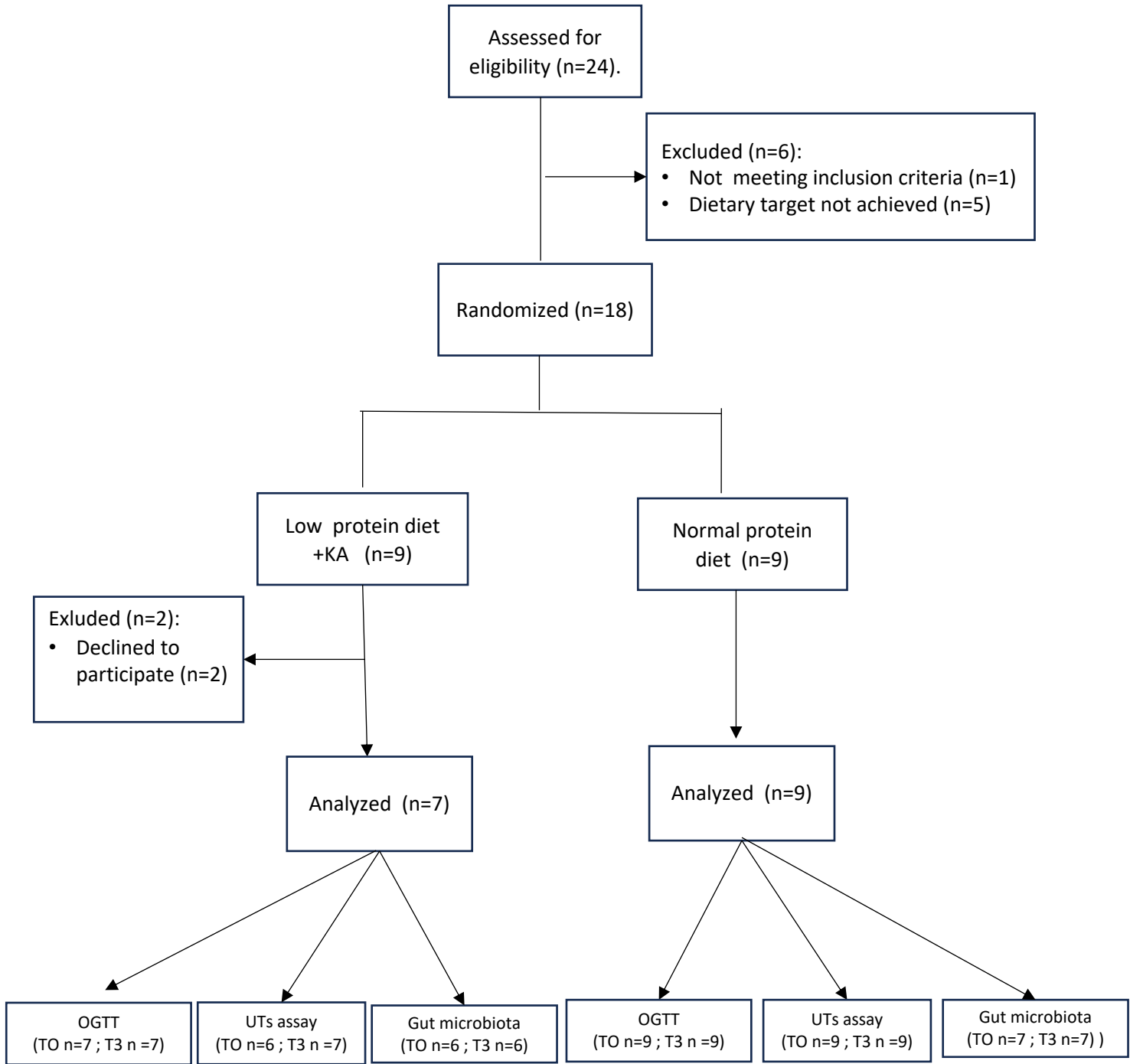

Supplement: Supplementary Material — Fig_S1.pdf [file KGMI_A_2696622_SM1926.pdf]

Figure S2

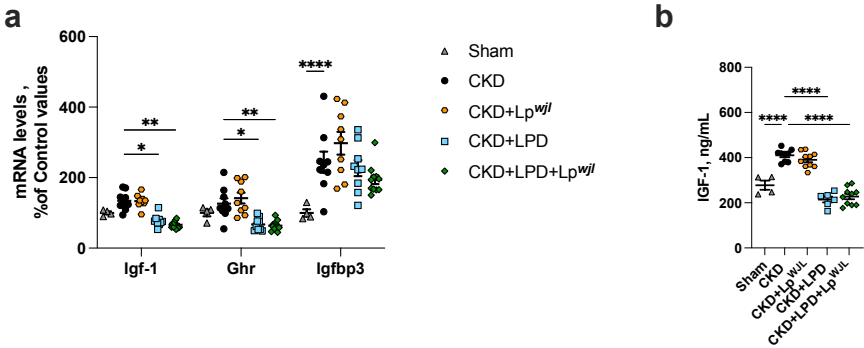

Supplement: Supplementary Material — Fig_S2.pdf [file KGMI_A_2696622_SM1815.pdf]

a

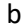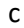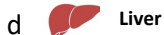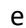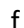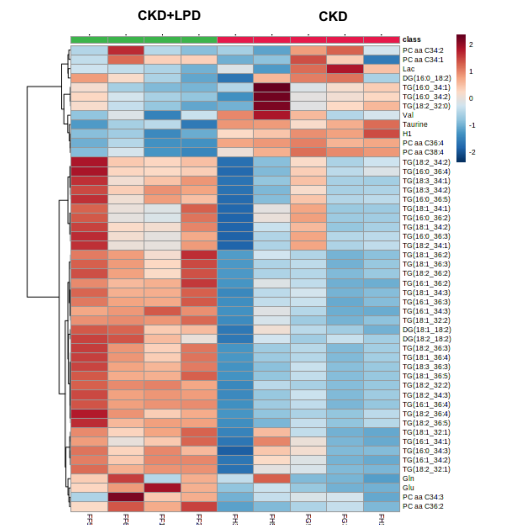

Supplement: Supplementary Material — Fig_S3.pdf [file KGMI_A_2696622_SM1656.pdf]

Figure S4

a

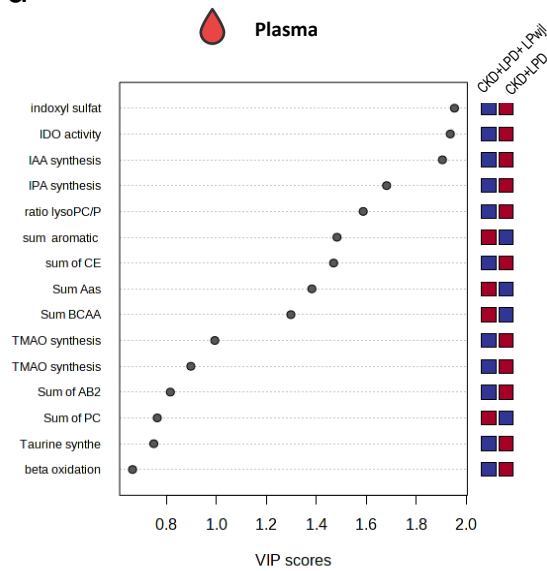

b

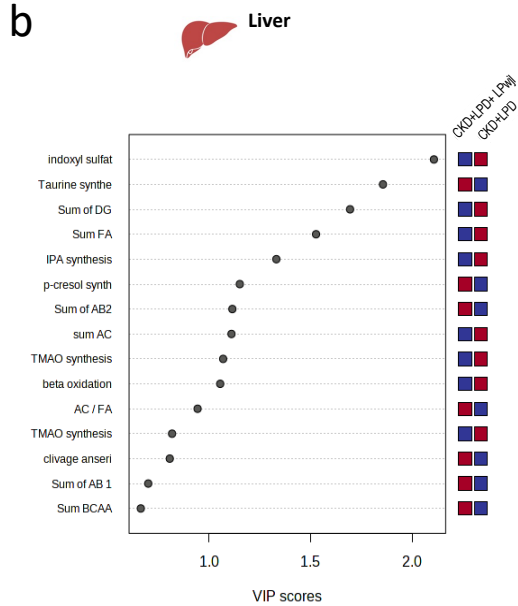

c

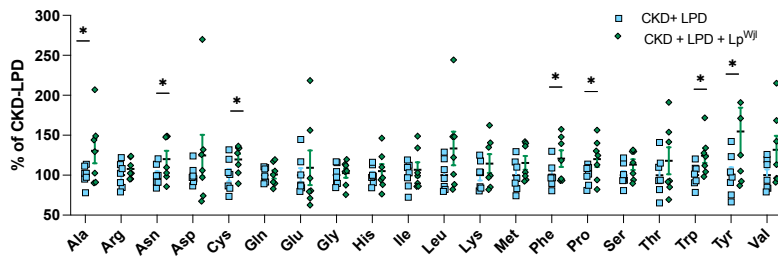

Supplement: Supplementary Material — Fig_S4.pdf [file KGMI_A_2696622_SM1536.pdf]

Figure S5

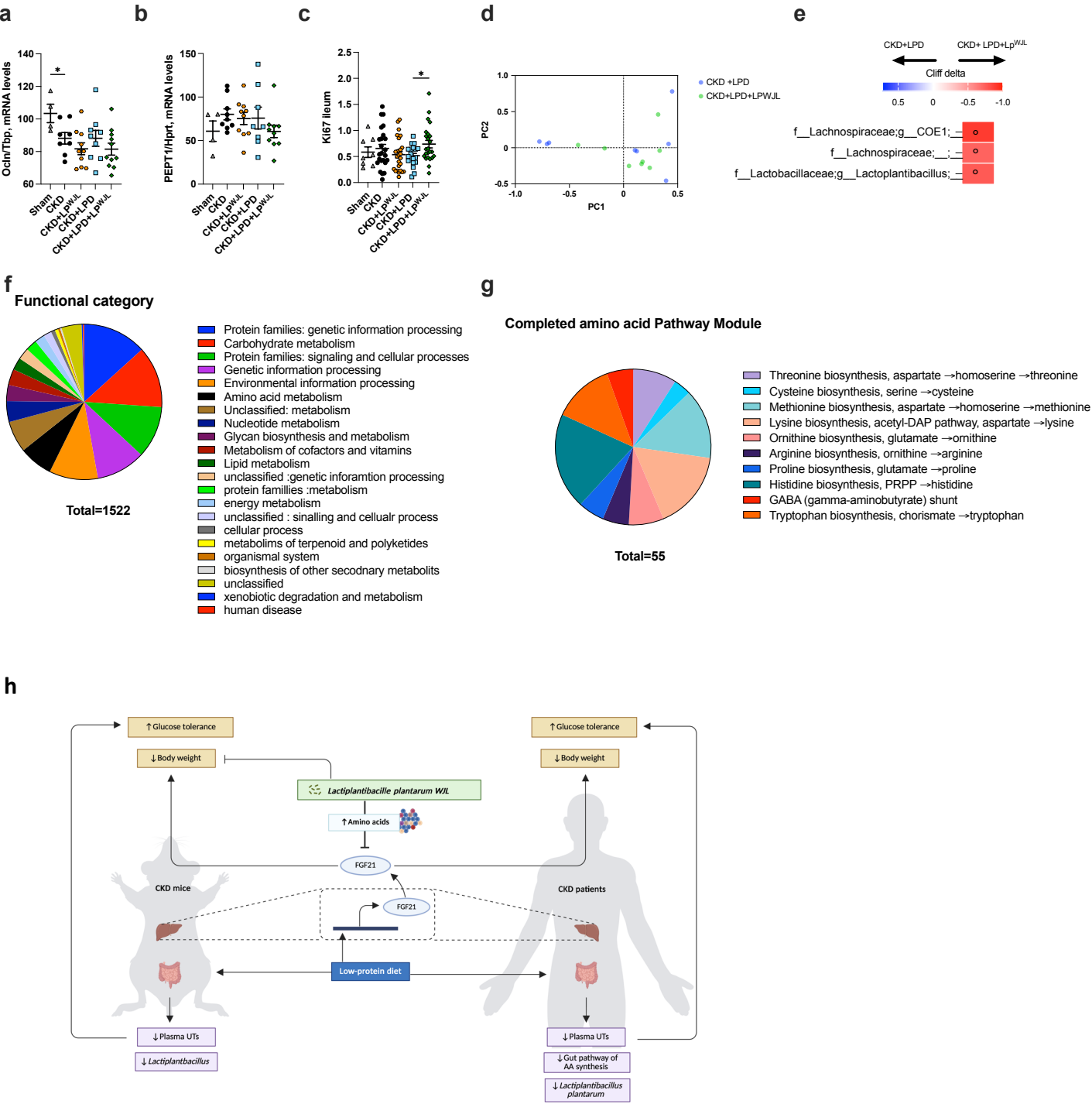

Supplement: Supplementary Material — Fig_S5.pdf [file KGMI_A_2696622_SM1388.pdf]
