# Supplementary material for: Lactiplantibacillus plantarum WJL ameliorates chronic kidney disease by inhibiting fibroblast growth factor 21 adaptive stress response via low protein diet
Source: Gut Microbes. 2026 Jul 12;18(1):2696622. doi: 10.1080/19490976.2026.2696622 (PMC13367091; doi:10.1080/19490976.2026.2696622)
Supplement: Supplementary Material — ARRIVEchecklistVf.docx [file KGMI_A_2696622_SM1369.docx]

# Reporting checklist for study using laboratory animals.

Based on the ARRIVE guidelines.

## Instructions to authors

Complete this checklist by entering the page numbers from your manuscript where readers will find each of the items listed below.

Your article may not currently address all the items on the checklist. Please modify your text to include the missing information. If you are certain that an item does not apply, please write "n/a" and provide a short explanation.

Upload your completed checklist as an extra file when you submit to a journal.

In your methods section, say that you used the ARRIVEreporting guidelines, and cite them as:

Percie du Sert N, Hurst V, Ahluwalia A, Alam S, Avey MT, Baker M, Browne WJ, Clark A, Cuthill IC, Dirnagl U, Emerson M, Garner P, Holgate ST, Howells DW, Karp NA, Lazic SE, Lidster K, MacCallum CJ, Macleod M, Pearl EJ, Petersen O, Rawle F, Peynolds P, Rooney K, Sena ES, Silberberg SD, Steckler T and Wurbel H. The ARRIVE Guidelines 2.0: updated guidelines for reporting animal research.

|  |  | Reporting Item | Page Number |
| --- | --- | --- | --- |
| **Essential 10** |  |  |  |
| Study design | [#1a](https://www.goodreports.org/reporting-checklists/arrive2/info/#1a) | Give details of the groups being compared, including control groups. If no control group has been used, the rationale should be stated. | Supplementary Files  Page 1-2 |
| Study design | [#1b](https://www.goodreports.org/reporting-checklists/arrive2/info/#1b) | Give details of the experimental unit (e.g., a single animal, litter, or cage of animals). | Supplementary Files  Page 1-2 |
| Sample size | [#2a](https://www.goodreports.org/reporting-checklists/arrive2/info/#2a) | Specify the exact number of experimental units allocated to each group, and the total number in each experiment. Also indicate the total number of animals used. | Supplementary Files  Page 1-2 |
| Sample size | [#2b](https://www.goodreports.org/reporting-checklists/arrive2/info/#2b) | Explain how the sample size was decided. Provide details of any a priori sample size calculation, if done. | Supplementary Files  Page 2 |
| Inclusion and exclusion criteria | [#3a](https://www.goodreports.org/reporting-checklists/arrive2/info/#3a) | Describe any criteria used for including or excluding animals (or experimental units) during the experiment, and data points during the analysis. Specify if these criteria were established a priori. If no criteria were set, state this explicitly. | Supplementary Files  Page 1-3 |
| Inclusion and exclusion criteria | [#3b](https://www.goodreports.org/reporting-checklists/arrive2/info/#3b) | For each experimental group, report any animals, experimental units, or data points not included in the analysis and explain why. If there were no exclusions, state so. | See in legend figures page 24 to 33 in main manuscript |
| Inclusion and exclusion criteria | [#3c](https://www.goodreports.org/reporting-checklists/arrive2/info/#3c) | For each analysis, report the exact value of n in each experimental group. | See in legend figures page 25 to 33 in main manuscript |
| Randomisation | [#4a](https://www.goodreports.org/reporting-checklists/arrive2/info/#4a) | State whether randomisation was used to allocate experimental units to control and treatment groups. If done, provide the method used to generate the randomisation sequence. | Supplementary Files  Page 1-2 |
| Randomisation | [#4b](https://www.goodreports.org/reporting-checklists/arrive2/info/#4b) | Describe the strategy used to minimise potential confounders such as the order of treatments and measurements, or animal/cage location. If confounders were not controlled, state this explicitly. | Supplementary Files  Page 1-2 |
| Blinding | [#5](https://www.goodreports.org/reporting-checklists/arrive2/info/#5) | Describe who was aware of the group allocation at the different stages of the experiment (during the allocation, the conduct of the experiment, the outcome assessment, and the data analysis). | NA |
| Outcome measures | [#6a](https://www.goodreports.org/reporting-checklists/arrive2/info/#6a) | Clearly define all outcome measures assessed (e.g., cell death, molecular markers, or behavioural changes). | Page 10 in main manuscript |
| Outcome measures | [#6b](https://www.goodreports.org/reporting-checklists/arrive2/info/#6b) | For hypothesis-testing studies, specify the primary outcome measure, i.e., the outcome measure that was used to determine the sample size. | NA |
| Statistical methods | [#7a](https://www.goodreports.org/reporting-checklists/arrive2/info/#7a) | Provide details of the statistical methods used for each analysis, including software used. | Supplementary Files  Page 18 |
| Statistical methods | [#7b](https://www.goodreports.org/reporting-checklists/arrive2/info/#7b) | Describe any methods used to assess whether the data met the assumptions of the statistical approach, and what was done if the assumptions were not met. | NA |
| Experimental animals | [#8a](https://www.goodreports.org/reporting-checklists/arrive2/info/#8a) | Provide species-appropriate details of the animals used, including species, strain and substrain, sex, age or developmental stage, and, if relevant, weight. | Supplementary Files  Page 1-2 |
| Experimental animals | [#8b](https://www.goodreports.org/reporting-checklists/arrive2/info/#8b) | Provide further relevant information on the provenance of animals, health/immune status, genetic modification status, genotype, and any previous procedures. | Supplementary Files  Page 1-2 |
| Experimental procedures | [#9a](https://www.goodreports.org/reporting-checklists/arrive2/info/#9a) | For each experimental group, including controls, describe the procedures in enough detail to allow others to replicate what was done, how it was done, and what was used. | Supplementary Files  Page 1-2 |
| Experimental procedures | [#9b](https://www.goodreports.org/reporting-checklists/arrive2/info/#9b) | Timing and frequency of procedures | Supplementary Files  Page 1-2 and Fig 1a and 5aa |
| Experimental procedures | [#9c](https://www.goodreports.org/reporting-checklists/arrive2/info/#9c) | Where procedures were carried out (including detail of any acclimatisation periods). | Supplementary Files  Page 1-2 |
| Experimental procedures | [#9d](https://www.goodreports.org/reporting-checklists/arrive2/info/#9d) | Rationale for procedures | Supplementary Files  Page 1 |
| Results | [#10a](https://www.goodreports.org/reporting-checklists/arrive2/info/#10a) | For each experiment conducted, including independent replications, report summary/descriptive statistics for each experimental group, with a measure of variability where applicable (e.g., mean and SD, or median and range). | See in legend figures page 25 to 33 in main manuscript |
| Results | [#10b](https://www.goodreports.org/reporting-checklists/arrive2/info/#10b) | If applicable, for each experiment conducted, including independent replications, report the effect size with a confidence interval. | NA |

None The ARRIVE checklist is distributed under the terms of the Creative Commons Attribution License CC-BY. This checklist can be completed online using <https://www.goodreports.org/>, a tool made by the [EQUATOR Network](https://www.equator-network.org) in collaboration with [Penelope.ai](https://www.penelope.ai)
