# Supplementary material for: Lactiplantibacillus plantarum WJL ameliorates chronic kidney disease by inhibiting fibroblast growth factor 21 adaptive stress response via low protein diet
Source: Gut Microbes. 2026 Jul 12;18(1):2696622. doi: 10.1080/19490976.2026.2696622 (PMC13367091; doi:10.1080/19490976.2026.2696622)
Supplement: Supplementary Material — Supplementary_Figure.docx [file KGMI_A_2696622_SM8466.docx]

**Supplementary Figure:**

**Figure S1. CONSORT flow chart of the clinical study.**

**Figure S2. *Lactiplantibacillus plantarum* WJL does not modulate the GH/IGF-1 axis in CKD mice under standard or low-protein diets.**

(a) Hepatic mRNA expression levels of insulin-like growth factor 1 (*Igf1*), growth hormone receptor (*Ghr*), and insulin-like growth factor binding protein 3 (*Igfbp3*), normalized to TATA-box binding protein (*Tbp*) and expressed relative to sham mice (n = 4 for Sham, n = 9 for CKD, n = 9 for CKD + LP^WJL^, n=9 for CKD +LPD, n=11 for CKD+LPD+ Lp^WJL^). (b) Plasma IGF-1 concentration (n = 4 for Sham, n = 9 for CKD, n = 10 for CKD + LP^WJL^, n=6 for CKD +LPD, n=10 for CKD+LPD+ Lp^WJL^). Data are presented as mean ± SEM. Statistical analysis was performed using one-way ANOVA followed by Bonferroni post hoc test. *p < 0.05, **p < 0.01, ***p < 0.001, ****p < 0.0001.

**Figure S3: Low-protein diet alters blood and liver metabolomes in CKD mice.**

(a, d) Variable importance in projection (VIP) plots showing the top 25 metabolites (VIP > 1.8) discriminating groups in (a) plasma (n = 7 for CKD, n=7 for CKD +LPD, n=7 for CKD+LPD+ Lp^WJL^) and (d) liver samples (n = 5 for CKD, n=5 for CKD +LPD, n=5 for CKD+LPD+ Lp^WJL^), based on partial least squares discriminant analysis (PLS-DA) using MetaboAnalyst comparing CKD vs. CKD-LPD groups. (b, e) Metabolite set enrichment analysis (MSEA) of identified metabolites mapped to KEGG human metabolic pathways in (b) plasma and (e) liver, comparing CKD vs. CKD-LPD groups. (c, f) Heatmaps showing relative abundance of detected metabolites in (c) plasma and (f) liver samples. Color scale represents normalized intensity values ranging from blue (low abundance) to red (high abundance) comparing CKD vs. CKD-LPD group.

**Figure S4. Ratios of metabolic pathway indicators and plasma amino acid concentrations in CKD mice under low-protein diet with or without *Lactiplantibacillus plantarum* WJL supplementation.**

(a–b) Variable importance in projection (VIP) score plots showing the top 15 metabolic indicators (VIP > 0.8) derived using MetaboINDICATOR™, a tool that aggregates metabolites into functional ratios and sums, based on partial least squares discriminant analysis (PLS-DA) comparing CKD mice under low-protein diet (LPD) versus LPD supplemented with *Lactiplantibacillus plantarum* WJL (Lp^WJL^). Data are shown for (a) plasma plasma (n = 7 for CKD, n=7 for CKD +LPD, n=7 for CKD+LPD+ Lp^WJL^) and (b) liver (n = 5 for CKD, n=5 for CKD +LPD, n=5 for CKD+LPD+ Lp^WJL^). (c) Relative plasma concentrations of amino acids in CKD mice under LPD and LPD + Lp^WJL^ plasma (n = 7 for CKD, n=7 for CKD +LPD, n=7 for CKD+LPD+ Lp^WJL^). Data are presented as mean ± SEM. Statistical analysis was performed using one-way ANOVA followed by Bonferroni post hoc test

**Figure S5. Impact of *Lactiplantibacillus plantarum* WJL on gut barrier markers and microbiota structure in CKD mice under low-protein diet or standard diet.**

(a) Ileal mRNA expression of occludin (Ocln) (n = 4 for Sham, n = 8 for CKD, n = 10 for CKD + LP^WJL^, n=9 for CKD +LPD, n=11 for CKD+LPD+ Lp^WJL^). and (b) peptide transporter 1 (Pept1) (n = 4 for Sham, n = 9 for CKD, n = 10 for CKD + LP^WJL^, n=8 for CKD +LPD, n=10 for CKD+LPD+ Lp^WJL^), normalized to TATA-box binding protein (Tbp) and expressed relative to sham mice, in CKD mice receiving a standard or low-protein diet (LPD), with or without *Lactiplantibacillus plantarum* WJL (Lp^WJL^) supplementation. (n = 4 for Sham, n = 9 for CKD, n = 10 for CKD + LP^WJL^, n=6 for CKD +LPD, n=10 for CKD+LPD+ Lp^WJL^).. (c) Ileal proliferative activity assessed by quantification of Ki67-positive cells (n = 4 for Sham, n = 9 for CKD, n = 10 for CKD + LP^WJL^, n=9 for CKD +LPD, n=11 for CKD+LPD+ Lp^WJL^). .Data are presented as mean ± SEM. Statistical analysis was performed using one-way ANOVA followed by Bonferroni post hoc test.(d) Principal coordinates analysis (PCoA) based on Bray–Curtis dissimilarity (β-diversity), comparing CKD mice under LPD alone or LPD supplemented with Lp^WJL^. (n=8 for CKD +LPD, n=8 for CKD+LPD+ Lp^WJL^). (e) Heatmap scaled to Cliff’s delta effect size at the species level, showing taxa identified by 16S rRNA sequencing as significantly enriched in CKD + LPD or CKD + LPD + Lp^WJL^ (p < 0.05, Cliff’s delta > 0.1). Taxonomic annotations: g_ for genus, and f_ for family. False discovery rates (q-values) were calculated using the Mann–Whitney test with Benjamini–Hochberg correction. Circles indicate q < 0.1. Blue indicates enrichment in CKD + LPD, red indicates enrichment in CKD + LPD + Lp^WJL^. (f) Functional annotation of Lp^WJL^ genome based on KEGG orthology.(g) Amino acid metabolism subsystem feature counts from whole-genome sequencing of Lp^WJL^. (i) Schematic representation of the impact of low protein diet and Lp^WJL^ supplementation in CKD.
