# Supplementary material for: Lactiplantibacillus plantarum WJL ameliorates chronic kidney disease by inhibiting fibroblast growth factor 21 adaptive stress response via low protein diet
Source: Gut Microbes. 2026 Jul 12;18(1):2696622. doi: 10.1080/19490976.2026.2696622 (PMC13367091; doi:10.1080/19490976.2026.2696622)
Supplement: Supplementary Material — Supplementary clean version .docx [file KGMI_A_2696622_SM6181.docx]

**Supplementary methods**

**Experimental Model**

**Study Approval**

All animal procedures were performed in compliance with institutional and European regulations for animal experimentation. The protocol was approved by the French Ministry of Agriculture (Decree n°2013-118), the European Union Directive 2010/63/EU, and the local ethics committee (reference #2021112217245161 and #2022080515563596). All experiments are conformed with the ARRIVE guidelines

**Mice**

Male C57BL/6JRj mice (4–6 weeks old) were purchased from ENVIGO RMS SARL (Gannat, France). Animals were housed in the specific pathogen free animal facility at the *Plateau de Biologie Expérimentale*, *École Normale Supérieure de Lyon*, Lyon, France. Mice were group-housed (5 per cage) under controlled conditions (21 ± 0.5°C, 60–70% humidity, 12h light/dark cycle). All animals had ad libitum access to water and standard chow, and were given two weeks for acclimatization. Daily health checks were performed by the veterinary staff. Food and water intakes were measured twice weekly. The daily food and water intakes were calculated for each cage as the difference between the amount given and that removed from the cage. Body composition was assessed using a Minispec LF50 Body Composition Analyzer (Bruker, Billerica, MA, USA) before necropsy.

**Chronic kidney disease (CKD) models, diets, and treatments**

Chemical nephrectomy was induced with a 0.25% (w/w) adenine-enriched A04 diet (SAFE, Augy, France) for 4 weeks in 20 mice. After a 2-week washout, mice were randomized to receive either a LPD (6.1 % total protein, AIN-93G formulation, INRAE, Lyon, France) or continue on standard diet for 6 weeks (18.8 % total protein, AIN-93G formulation). A control group (n=10) received standard diet (AIN93G) during all the procedure (Fig. 1a, Table S1). We planned to use 10 animals per group in the CKD model to detect a 10% difference in fibrosis, based on our previous observations^1^. No mortality was expected by this diet. Three groups were defined: Control (n=10), CKD (n=10), and CKD+LPD (n=10). The mice were housed in groups of four to five animals per cage. All animals were analyzed.

The 5/6 nephrectomy (5/6 Nx) model involved two surgical steps: electrocoagulation of upper and lower poles of the right kidney, followed one week later by removal of the left kidney after vessel and ureter ligation. Surgeries were performed under general anesthesia (ketamine 100 mg/kg, Imalgène 1000, MERIAL, Lyon, France; xylazine 20 mg/kg, Rompun 2%, CENTRAVET, Loudéac, France). Sham mice underwent surgery without kidney removal. Postoperative analgesia consisted in buprenorphine (0.05 mg/kg, Axience, Pantin, France) given i.p. 3 times/day for 2 days. One week post-surgery, mice were randomized into 5 groups receiving either the probiotic Lp^WJL^ (3.10⁸ CFU in 20% maltodextrin + 2% dextrose) or vehicle control. Probiotic and vehicle were provided by the *Institut de Génomique Fonctionnelle de Lyon* (Lyon, France). Treatment was administered by oral gavage 5 times per week. Each group received either standard (18.8%) or LPD (6.1%; Table S1). We planned to use 10 animals per group in the CKD model to detect a 10% difference in fibrosis, based on our previous observations.^1^ Considering an expected mortality rate of 10% observed in our prior experiments, we initially included 12 mice per CKD group. 4 mice were included in the control group.3 mice died in the CKD group, 2 mice in the CKD +Lp^WJL^ group, 3 mice in the CKD + LPD group, and 1 mouse in the CKD +LPD + Lp^WJL^. All deaths occurred immediately after surgery, and these animals were not included in the analysis. Groups included: Sham (n=4), CKD (n=9), CKD+Lp^WJL^ (n=.10), CKD+LPD (n=9), and CKD+LPD+Lp^WJL^ (n=11; Fig. 5a). The mice were housed in groups of four to five animals per cage. All animals were analyzed.

**Tissues and blood collection and biochemical measurements**

After 6 weeks of diet, mice were placed in metabolic cages (Charles River, Ecully, France) for 24 h urine collection. Urine volume was determined by weight. Proteinuria was assessed by Bradford assay (Sigma-Aldrich, Saint-Quentin-Fallavier, France); albumin by ELISA (Bethyl Laboratories, Montgomery, TX, USA). At endpoint, mice were euthanized (ketamine/xylazine 100/20 mg/kg i.p.). Blood was collected from the left ventricle (EDTA-coated syringe), centrifuged (8 min, 10,000g) and plasma stored at −80°C. Kidneys, gastrocnemius, eWAT, liver, heart, caecum, and ileum were dissected, snap-frozen in liquid nitrogen, or fixed in 4% paraformaldehyde. Urea was quantified using a commercial kit (Cayman, Ann Arbor, MI, USA), and insulin by ELISA (Crystal Chem, Elk Grove Village, IL, USA). IgF1 was measured by ELISA (ThermoFisher Scientific, Waltham, MA, USA).

**Metabolic Studies**

Intraperitoneal Glucose Tolerance Test (i.p.GTT): after a 5-hour fast, mice were injected intraperitoneally with D-glucose (2 g/kg) dissolved in sterile water (Sigma-Aldrich, Saint-Quentin-Fallavier, France). Blood glucose was measured at baseline (0 min) and at 15, 30, 45, 60, and 90 minutes post-injection. Glucose levels were determined from a drop of blood collected from the tail tip using a glucometer (Accu-Chek, Roche Applied Science, Bâle, Switzerland). The area under the curve (AUC), corrected for baseline glycemia, was calculated.

Insulin Tolerance Test (ITT): after a 5-hour fast, mice were injected intraperitoneally with 0.75 U/kg of recombinant human insulin (Actrapid®, Novo Nordisk, Bagsværd, Denmark). Blood glucose levels were measured at 0 (baseline), 15, 30, 40, and 60 minutes after injection. The glucose disappearance rate (kITT; %/min) was calculated using the formula: kITT = 0.693 × 100 / t½, where t½ represents the half-life of plasma glucose concentration, estimated from the exponential decay slope over the first 30 minutes post-injection.

Homeostasis Model Assessment (HOMA): The HOMA-IR index was calculated using the formula: HOMA-IR = (fasting glucose [mmol/L] × fasting insulin [μU/mL]) / 22.5.

**Measurement of uremic toxins**

Mice total plasmatic levels of uremic toxins (UTs) were quantified by ultra-performance liquid chromatography with ultraviolet and fluorescence detection (UPLC-UV/FLD) as previously described.^2^

**Renal Histology**

Kidneys were fixed in 4% paraformaldehyde, paraffin-embedded, and sectioned at 4 µm thickness. Sections were stained with Sirius Red (Sigma-Aldrich, Saint-Quentin-Fallavier, France) to assess interstitial fibrosis. Sirius Red-stained sections were examined under polarization contrast illumination using consistent camera settings of the Leica Thunder Imager. We maintained the lamp intensity, camera exposure settings, and camera gain at constant levels, considering the placement of orthogonal polarizing filters and ensuring image focus. Sirius Red fibrosis was quantified using whole kidney sections from tiled 10× magnification images using an Olympus BX63 microscope (Olympus France, Rungis, France). For each mouse, 3 full kidney sections were analyzed, with 10 to 20 non-overlapping fields imaged per section. Sham-operated mice typically yielded 18 fields (6 per section), while CKD mice yielded 10 fields due to smaller kidney size. For the quantification of fibrosis area, we adapted a script in Fiji, which was kindly provided by the author of a previously published method.^3^ ^4^ The region of interest (ROI) within the kidney section was manually outlined, excluding the capsule, internal and external medullary regions. To calculate the percentage of fibrosis within this ROI, we followed a three-step process: 1) we used the intensity distribution to establish a background intensity threshold; 2) peri-vascular regions were excluded from the ROI using a Gaussian filtering and thresholding technique; and 3) the percentage of fibrosis was determined as the proportion of unmasked pixels above the established threshold, relative to the total number of pixels within the ROI.

**Intestinal Histology and Ki67 Quantification**

Ileal tissue was fixed in 4% paraformaldehyde, paraffin-embedded, and sectioned at 4 µm. Sections were subjected to immunohistochemical staining for Ki67 (a marker of cell proliferation). After deparaffinization and rehydration, antigen retrieval was performed in citrate buffer (10 mM, pH 6.0) at 95°C for 20 min. Sections from the segments of ileum were blocked with 3% BSA and incubated overnight at 4°C with rabbit anti-Ki67 (1:100, AbCam, ab15580). Detection was achieved using HRP-conjugated secondary antibodies and DAB substrate. Counterstaining was performed with hematoxylin. Ki67-positive cells were counted in 10 randomly selected non-overlapping crypts per section and expressed as a percentage of total epithelial cells.

**Gene Expression Analysis**

Total RNA was extracted from frozen tissue samples using TRIzol reagent (ThermoFisher Scientific, Illkirch, France) according to the manufacturer’s instructions. RNA concentration and purity were assessed using a NanoDrop 2000 spectrophotometer (ThermoFisher Scientific). For each sample, 1 μg of RNA was reverse-transcripted into cDNA using a high-capacity cDNA reverse transcription kit. Quantitative real-time PCR was performed using the QuantiTect SYBR Green PCR Kit (Qiagen, Mississauga, ON, Canada) on a StepOnePlus Real-Time PCR System (Applied Biosystems, Foster City, CA, USA). Gene expression levels were normalized to the housekeeping gene TATA-box binding protein (TBP), and results were expressed as fold changes relative to the mean expression levels in sham-operated mice. Primer sequences used for target gene amplification are listed in Supplementary Table S10.

**DNA Extraction, 16S rRNA Sequencing, and Microbiota Data Analysis in mice**

Cecal contents were processed for bacterial 16S rRNA gene sequencing by the ProfilExpert platform (Lyon, France). DNA was extracted from 15 mg of feces using the Zymobiomics DNA Microprep Kit (Zymo Research, Irvine, CA, USA; Ref #D4301) following the manufacturer’s instructions. A total of 40 ng of purified DNA per sample was used for library preparation targeting the V3–V4 hypervariable regions of the 16S rRNA gene, using the Quick-16S NGS Library Prep Kit (Ozyme, Saint-Cyr-l'École, France). Sequencing was performed on an Illumina MiSeq platform using the MiSeq Reagent Kit v3 (600-cycle). Raw sequencing reads were demultiplexed using Bcl2fastq software (v2.17.1.14) and trimmed with cutadapt (v1.9.1) to remove adapter sequences and low-quality bases. Amplicon sequence variants (ASVs) were inferred using the DADA2 algorithm implemented in QIIME2 (Quantitative Insights Into Microbial Ecology,), employing a zero-noise operational taxonomic unit (OTU) approach. Taxonomic assignment of ASVs was performed using the Greengenes reference database (version 13.8, 99% OTUs).

**Metabolomic Analyses**

Frozen liver samples (100 mg) were transferred into 2 mL Precellys tubes containing 1.4 mm ceramic beads. Samples were homogenized with 300 µL of isopropanol using a Precellys 24 homogenizer (Bertin Technologies, Montigny-le-Bretonneux, France) for three 30-second cycles at 5,800 rpm with 30-second intervals. Homogenates were centrifuged at 10,000 g for 5 minutes at 4 °C, and supernatants were collected and stored at −80 °C until analysis. Sample preparation (using 10 µL serum), instrument analyses, quality control measures and checks, and metabolite quantification were performed in accordance with the manufacturer’s instructions. All metabolomics analyses were performed at the METANUTRIBIOTA Metabolomics Platform (Lyon, France). Serum samples were analyzed using the MxP® Quant 500 kit (Biocrates Life Sciences AG, Innsbruck, Austria; https://biocrates.com/mxp-quant-500-kit/; accessed February 22, 2024), on a XEVO TQ-XS triple quadrupole mass spectrometer coupled to an Acquity UPLC system (Waters Corporation, Milford, MA, USA). This targeted assay quantifies up to 630 metabolites spanning 26 biochemical classes using both LC-MS/MS and FIA-MS/MS methodologies. All procedures followed the manufacturer’s protocol without deviation. Reagents, columns, and internal standards were those supplied with the kit. LC and FIA methods operated in both positive and negative ionization modes with predefined instrument-specific settings. Sample preparation involved derivatization, extraction, and dilution on 96-well filter plates preloaded with internal standards. External calibration standards and quality controls were included as recommended. Metabolite classes analyzed included alkaloids, amine oxides, amino acids, amino acid-related metabolites, bile acids, biogenic amines, carboxylic acids, cresols, fatty acids, hormones, indole derivatives, nucleobase-related metabolites, vitamins, cofactors, acylcarnitines, lysophosphatidylcholines, phosphatidylcholines, sphingomyelins, ceramides, and various glycosylated ceramides, diglycerides, triglycerides, and cholesteryl esters. Quantification was achieved using a seven-point calibration curve for LC-MS/MS or a single-point calibration for FIA-MS/MS, with isotopically labeled internal standards or class-specific surrogates, as predefined in the kit's quantification software. Data acquisition was performed using MassLynx® software (Waters), and data analysis was completed with WebIDQ™ software (Biocrates Life Sciences AG), which also enabled calculation of over 200 MetaboINDICATOR™ metrics (metabolite sums and ratios) to facilitate interpretation.

**Human study**

**Study Design and Participants**

The KETO-GUT study was an open-label, randomized, controlled clinical trial designed to evaluate the effects of a low-protein diet supplemented with ketoanalogues (LPD+KA) compared to a normal protein diet (ND) in patients with advanced non-diabetic chronic kidney disease (CKD) on UTs levels, gut microbiota composition, and metabolic parameters. Participants were randomly assigned in a 1:1 ratio to receive either a ND (0.8 g/kg/day) or a LPD (0.4 g/kg/day) supplemented with KA (1 tablet per 5 kg body weight per day). Randomization was performed using a computer-generated sequence with permuted blocks. All participants provided written informed consent before any study procedure. The study was approved by the institutional review board *CPP Ouest II-Angers* (reference RCB 2019-02225-30) and was registered on ClinicalTrials.gov (NCT03959228).

**Recruitment**

Participants were recruited from the Nephrology Department of Hôpital Lyon Sud (Hospices Civils de Lyon). From September 2019 to June 2024, all patients aged between 18 and 80 years, with an estimated glomerular filtration rate (eGFR) <30 mL/min/1.73 m² and without diabetes, were systematically screened. Patients who expressed interest received study information and eligibility was confirmed by the principal investigator.

**Inclusion and Exclusion Criteria**

Eligible participants met the following criteria: 1) age between 18 and 80 years; 2) diagnosed with stage 4 or 5 CKD (eGFR <30 mL/min/1.73 m²); 3) BMI between 18 and 33 kg/m²; and 4) no diagnosis of diabetes (fasting plasma glucose ≥ 1.26 g/L (7.0 mmol/L), HbA1c ≥ 6.5%, or treatment with glucose-lowering medications). Exclusion criteria included: current dialysis; history of kidney transplantation; pregnancy or breastfeeding; refusal or inability to comply with dietary counselling; severe malnutrition (serum albumin <38 g/L); presence of active inflammatory, infectious, cardiovascular, or neoplastic disease; prior large bowel resection; medically confirmed irritable bowel syndrome (IBS) or inflammatory bowel disease (IBD); ; treatment with more than two grams of calcium per day; use of laxatives more than two doses per day; uncontrolled metabolic acidosis with serum bicarbonate below 18 mmol/L; hyperparathyroidism defined as parathyroid hormone greater than five times the upper limit of normal; hypercalcemia greater than 2.55 mmol/L or hypophosphatemia below 0.70 mmol/L; anemia with hemoglobin below 80 g/dl, antibiotic, probiotic, or prebiotic use within one month before inclusion; known hypersensitivity to any component or excipient of KA, significant modification of immunosuppressive therapy within six months prior to study entry; absence of national health insurance; and intellectual, cognitive, or hearing impairment interfering with study participation.

**Covariates**

Demographic characteristics, medical history and treatment were self-reported by participants using a standardized questionnaire and using medical records. Cardiovascular disease (CVD) was defined as a history of physician-diagnosed myocardial infarction, stroke, resuscitated cardiac arrest, heart failure, or prior coronary or cerebral revascularization procedures (e.g., angioplasty, bypass surgery, carotid endarterectomy).

**Intervention**

Regardless of group allocation, all participants received individualized dietary education in accordance with evidence-based guidelines for the nutritional management of CKD available at the time the study was initiated.^5^ The dietary plan was tailored to each participant’s specific nutritional needs, food preferences, culinary skills, and access to cooking facilities. It ensured adequate macro- and micronutrient intake and accounted for relevant comorbidities or nutrition-related concerns, such as fluid and sodium balance or lipid management. During the 3-month run-in period preceding randomization, the dietary objective for all participants was to achieve a protein intake of 0.8 g/kg/day. A baseline dietary consultation was conducted by an experienced nephrology dietitian at inclusion, followed by monthly assessments. Protein intake was estimated from 24-hour urinary urea excretion using the Maroni formula.^6^ Urea was measured in mmol/L and converted to urea nitrogen (g/day) using the standard molecular weight ratio (urea nitrogen [g/day] = urea [mmol/day] × 0.028). The protein intake (g/day) was then calculated using the following formula: Protein intake (g/day) = (urinary urea nitrogen [g/day] + 0.031 × body weight [kg]) × 6.25. Only participants who achieved the target range by the end of the run-in phase (i.e; 0,8 g/kg/day ± 15 %) were eligible for randomization.

Participants were then randomized into two groups for a 3-month intervention period:

-Intervention group (LPD+KA): Participants received additional individualized dietary counselling aiming to reduce protein intake to 0.4 g/kg/day. This counselling was supported by written educational materials, including sample meal plans and protein equivalency tables commonly used in the nephrology unit. KA supplementation was prescribed at a dose of 1 tablet per 5 kg of body weight per day.

-Control group: Participants continued with the dietary guidance implemented during the run-in period, maintaining a target protein intake of 0.8 g/kg/day.

In both groups, protein intake was monitored monthly through 24-hour urine collections and recalculated using the Maroni formula.

At baseline (inclusion), randomization, and at the end of the intervention, participants completed a 3-day food record (including two weekdays and one weekend day) to corroborate urinary-based protein intake assessments. Sodium intake was estimated using 24-hour urinary sodium excretion, calculated as: Salt intake (g/day) = [urinary sodium (mmol/day)] / 17. Macronutrient (protein, lipids, carbohydrates, fibers) and caloric intake were calculated from dietary records using the French CIQUAL food composition database (ANSES, French Agency for Food, Environmental and Occupational Health Safety).

**Dietary adherence**

Protein intake was estimated monthly. If intake varied from the target range (±10% of the prescribed value), participants received an additional dietary counselling session, either by telephone or in person, to readjust their intake. These monthly assessments, alongside the 3-day food records and urinary urea analyses, were used to evaluate and monitor adherence to the assigned dietary intervention throughout the study.

**Oral Glucose Tolerance Test (OGTT)**

At randomization (T0) and 3 months later (T3), a standard 75 g oral glucose tolerance test (OGTT) was performed following an overnight fast of at least 10 hours. Participants were advised to avoid strenuous physical activity during the 24 hours preceding the test. On the morning of the test, individuals remained seated and at rest for 15 to 30 minutes prior to baseline measurements. At time 0 (baseline), a venous blood sample was collected to assess fasting serum glucose, insulin, and fibroblast growth factor 21 (FGF21) concentrations. Participants then ingested a solution containing 75 g of anhydrous glucose dissolved in 250–300 mL of water, consumed within 5 minutes under supervision. Subsequent venous blood samples were collected at 15, 30, 60, 90, 120, and 240 minutes after glucose ingestion. All samples were drawn from an antecubital vein using a butterfly catheter, and the first few milliliters were discarded to avoid dilution effects. Blood samples were allowed to clot at room temperature for 30 minutes before centrifugation at 4 °C for 10 minutes at 1,500 g. Serum and plasma were then aliquoted and stored at –80 °C until batch analysis. The total AUC for serum glucose, insulin, and FGF21 was calculated using the trapezoidal rule and corrected for baseline (fasting) values. AUCs were expressed in appropriate concentration-time units (mmol/L·min). Plasma glucose was measured using the enzymatic hexokinase method on a Cobas 8000 analyzer (Roche Diagnostics, France). Serum insulin was assessed via chemiluminescent microparticle immunoassay (Architect i2000SR, Abbott Diagnostics), and FGF21 was quantified using a validated ELISA kit (R&D Systems, USA; Cat# DF2100), following the manufacturer’s instructions.

**Biological Specimen Collection**

Biological specimens were collected longitudinally at the time of the randomization (T0) and after 3 months (T3) for each participant and included blood, urine, and fecal samples.

Fecal samples were collected by participants at home using standardized collection kits and returned under refrigerated conditions to BioMigen (France), where samples were processed according to standard operating procedures defined by the International Human Microbiome Standards Consortium SOP 5 (https://human-microbiome.org/index.php?id=Sop&num=005), funded by the European Commission. Samples were stored and processed for microbiome profiling on BioMigen’s analytical platform. 7 samples for the ND group at T0 and T3, and 6 samples for the LPD group at T0 and T3, were collected due to patients forgetting to send their samples within the study timeframe.

Urine samples consisted of 24-hour collections. Participants were instructed to discard the first morning void, then collect all subsequent urine over a 24-hour period, including the first void of the following morning, using dedicated collection containers. Samples were kept at 4°C during the collection period. Upon return to the biological center, total volume was measured, homogenized, and aliquoted. One aliquot was used for routine laboratory testing (urea, creatinine, electrolytes, protein).

Fasted blood samples were collected by a trained research nurse. Before the oral glucose tolerance test (OGTT), blood was drawn for both plasma and serum analysis. For plasma, blood was collected into EDTA tubes and centrifuged at 2,000 g for 15 minutes at 4 °C. For serum, blood was collected into serum-separator tubes (SST) and centrifuged at 1,500 g for 15 minutes at 4 °C. Five aliquots of both serum and plasma were prepared and stored at –80 °C. Additionally, at each OGTT time point (0, 15, 30, 60, 90, 120, and 240 minutes), one EDTA and one SST tube were collected, processed, and stored following the same protocol. One processed serum and plasma sample collected at baseline (prior to OGTT) was transported the same day to the clinical biochemistry laboratory at Hôpital Lyon Sud for routine analyses. This included serum creatinine and estimated glomerular filtration rate (eGFR) calculated using the CKD-EPI formula, urea, parathyroid hormone (PTH), bicarbonate, calcium, magnesium, phosphate, potassium, hemoglobin, ferritin albumin, prealbumin, and protein concentrations in blood. Furthermore, routine laboratory data from each participant’s local clinical laboratory were collected, at inclusion and also serum and urine parameters and 24-hour urinary urea as part of standard monthly monitoring.

**Anthropometric and Body Composition Measurements**

Body weight and composition (fat mass, fat-free mass, and hydration status) were assessed using the Body Composition Monitor (BCM, Fresenius Medical Care, Bad Homburg, Germany), a multifrequency bioelectrical impedance spectroscopy (BIS) device validated in patients with CKD. Measurements were performed under standardized conditions, with participants in a fasting state and after voiding, and recorded to the nearest 0.1 kg.

Height was measured using a wall-mounted stadiometer (Seca, Hamburg, Germany) while participants stood without shoes, and values were recorded to the nearest 0.1 cm. Body mass index (BMI) was calculated as weight in kilograms divided by height in meters squared (kg/m²).

Muscle strength was evaluated using a handgrip dynamometer (Jamar Hydraulic Hand Dynamometer, Patterson Medical, Warrenville, IL, USA). Measurements were taken on the dominant hand, with participants seated and the elbow flexed at 90°, according to standardized protocols. Three consecutive measurements were recorded, and the highest value (in kg) was retained for analysis.

**Blinding**

Due to the nature of the intervention, it was not possible to blind participants and investigators for outcome assessments after the first baseline visit. However, blinding of the allocation sequence was maintained during the analysis of all biological materials and those who cross-checked data entry.

**Sample Size Justification**

This study was exploratory. Given the limited data available at the time that investigated our outcomes of interest in adults with CKD, and to optimize both participant burden and study costs, prospective sample size estimations were conducted to determine an appropriate recruitment target. Notably, there was no study that integrated both a nutritional and microbiota-focused approach with mechanistic endpoints in a similar CKD population. Our study thus addresses a critical gap in the literature and provides important exploratory data in a field of unmet clinical and scientific need. Initially, based on recruitment capacity, logistical feasibility, and in light of the limited data available on serum UTs levels and gut microbiome composition in this patient population, a target of 50 participants was established. This sample size was considered sufficient to detect clinically relevant differences in both metabolic and microbiota-related endpoints, while allowing for anticipated dropouts. However, the recruitment phase coincided with the COVID-19 pandemic, which substantially disrupted patient flow and constrained clinical research activities in our nephrology department. Despite extending the inclusion period, we were ultimately unable to reach the projected recruitment target and successfully enrolled 24 participants.

**Uremic toxin measurement**

At randomization (T0) and 3 months later (T3), to determine total concentrations, 50 µL of serum samples were first precipitated with 340 µL of methanol after addition of 25 µL of internal standards. The supernatant obtained after 10 min centrifugation at 9000 g at 4°C was evaporated under nitrogen and the residue was reconstituted in 80 µL of water. Ultra-high-performance liquid chromatographic separation was performed on an Accucore PFP column (100 × 2.1 mm, 2.6 μm, Thermo, Les Ulis, France), and the detection on TSQ Quantiva tandem mass spectrometer (Thermo) operating in MRM mode. The method has been validated according to the European Medicines Agency (EMA) guidelines for method validation. Ten UTs (CMPF, hippuric acid, IAA, IS, kynurenic acid, kynurenine, PCG, PCS, PAG, and TMAO) and 3 precursors (tyrosine, phenylalanine, and tryptophan) in serum samples were quantified.^7^ The assay's limit of quantification was between 1 and 50 ng/mL, depending on the compound. The intra- and inter-day bias and CV (coefficient of variation) for the lower limit of quantification (LLOQ) were between −18.0% and 14.9%, and between 3.5% and 19.6%, respectively. Intra-day and inter-day bias and CV for quality control (QC) samples evaluated at 3 different concentrations (150, 8000, and 40 000 ng/mL) were all <13 % for the 13 assessed compounds.

**Fecal microbiota analysis in human**

Fecal samples were initially resuspended in 500 µL of SLX-Mus buffer (Omega Bio-tek, Norcross, GA, USA; Ref: SLXMLUS-1000). Genomic DNA was extracted from 50 µL of suspension using an internal protocol developed by BioMiGen. DNA concentration and purity were assessed by spectrophotometry, with quality control based on A260/280 and A260/230 ratios to ensure sample integrity. Extracted DNA was diluted 1:20 prior to amplification. The V3–V4 hypervariable regions of the bacterial 16S rRNA gene were amplified using a set of 36 primer pairs derived from 12 forward and 3 reverse primers, indexing each sample (Supplementary Table S11). Each primer pair targeted an amplicon of approximately 420 bp, and the final library size, including Illumina adapters and indices, was ~650 bp. PCR amplification was carried out using GoTaq® Rapid PCR Master Mix (Promega, Ref: CS3083A02) under the following thermal cycling conditions: initial denaturation at 95 °C for 1 minute, followed by 38 cycles of 95 °C for 4 seconds, 55 °C for 2 seconds, and 72 °C for 10 seconds, with a final elongation at 72 °C for 1 minute. PCR products were visualized and size-verified by capillary electrophoresis on a QIAxcel Advanced System (Qiagen) using a high-resolution DNA cartridge. Negative controls were included in each run and confirmed the absence of contamination. Amplicons were pooled in equimolar concentrations based on signal intensity and purified by gel electrophoresis on a 1.5% agarose gel using the PippinHT system (Sage Science; Marker 15C, Ref: 9789221832). Purified pooled libraries were quantified using Qubit 4.0 fluorometry (Thermo Fisher Scientific). Sequencing was performed on an Illumina MiSeq platform (Illumina, San Diego, CA, USA) using the MiSeq Reagent Kit v2 (2×251 bp, 500 cycles; Ref: MS-102-1003). Libraries were loaded at a theoretical cluster density of 800–1,000 K/mm². Demultiplexed reads were quality-checked using FastQC, and results were summarized with MultiQC. An initial abundance filter was applied to retain only the most representative taxa: bacterial species were included in the downstream analysis only if they represented at least 5% of the total abundance in at least one sample and were present in ≥30% of all samples. Functional inference of the microbiota was performed using PICRUSt2 (Phylogenetic Investigation of Communities by Reconstruction of Unobserved States; version 2.4.1; https://github.com/picrust/picrust2/archive/v2.4.1.tar.gz), which predicted metagenomic content based on the 16S rRNA gene sequences and generated enzyme classification numbers (EC numbers) for pathway analysis.

**CKD-MICROBIOME analysis:**

For the analysis of the correlation between gut microbiota composition and dietary protein intake, we used data from the prospective CKD MICROBIOME study embedded within the Chronic Kidney Disease Renal Epidemiology and Information Network cohort. This study characterized the gut microbiome of 240 non dialysis patients with CKD using whole genome shotgun metagenomic sequencing^8^, combined with detailed and standardized dietary intake recordings.^9^ The shotgun metagenomic sequencing datasets generated in this study have been deposited in the European Nucleotide Archive under accession number PRJEB81434 and are publicly accessible through the European Bioinformatics Institute platform (<https://www.ebi.ac.uk/ena/browser/view/PRJEB81434>).

**Whole-genome analysis of *Lactiplantibacillus plantarum WJL***

To infer the metabolic potential of Lp^WJL^, we retrieved its publicly available genome sequence (https://www.ncbi.nlm.nih.gov/nuccore/AUTE00000000) and submitted it to the GhostKOALA annotation server (https://www.kegg.jp/ghostkoala/) using the “genus_prokaryotes” database. The resulting KEGG Orthology (KO) assignments were used to reconstruct metabolic modules, focusing particularly on amino acid biosynthesis pathways. Enzyme Commission (EC) numbers and KEGG pathway maps were extracted to identify complete or partial metabolic pathways, with special attention to essential amino acids. Functional modules were curated manually based on the presence of core enzymatic steps required for each biosynthetic pathway.

**Statistical Analysis**

**Animal Studies**

For animal experiments, sample size was determined based on prior experience in similar models; no formal statistical method was used to predetermine group size. The number of independent experimental replicates performed for each experiment and the number of biological replicates for each experiment are indicated in figure legends. All quantitative results are presented as means ± standard error of the mean (SEM). For multiple group comparisons, one-way analysis of variance (ANOVA) was used, followed, when appropriate, by Bonferroni’s post hoc test for pairwise comparisons. For comparisons between two groups, a Student’s t-test was applied. Statistical significance was defined as p < 0.05.

**Metabolomic Analysis**

Metabolomic data were analyzed using MetaboAnalyst 6.0 (http://www.metaboanalyst.ca). Prior to analysis, all metabolite concentrations were autoscaled (mean-centered and divided by the standard deviation of each variable). Exploratory data analysis included principal component analysis (PCA) and partial least squares discriminant analysis (PLS-DA). Hierarchical clustering was conducted using Euclidean distance and Ward’s linkage method, with features visualized in heatmaps. Pathway enrichment and metabolite set enrichment analyses were also performed within the MetaboAnalyst framework.

**Gut microbiota**

Microbial community structure and diversity metrics were computed and visualized using QIIME2 (version 2020.8) with core analysis plugins. Alpha diversity was assessed using multiple indices, including Chao1 (species richness) and Shannon (diversity) metrics. Rarefaction was applied to normalize sequencing depth across all samples prior to diversity calculations. For visualization and downstream statistical analysis, Bray-Curtis distance matrices (beta diversity) were imported into R (version 4.0.3, released 2020-10-10) and analyzed using the phyloseq package (version 1.34.0). Functional inference based on 16S rRNA gene data, as well as taxonomic relative abundances, were down sampled and compared between groups. Group comparisons were conducted using non-parametric Wilcoxon–Mann–Whitney U tests. P-values were corrected for multiple testing using the Benjamini–Hochberg false discovery rate (FDR) method. Adjusted q-values < 0.1 were considered statistically significant. Effect sizes were reported using non-parametric directional standardized measures, including Cliff’s delta and Spearman’s rho for correlation analyses.

**Human studies**

The normality of continuous variables, expressed as raw values or as intra-individual differences between randomization (T0) and 3 months (T3), was assessed using the Shapiro–Wilk test. All subsequent statistical analyses were selected based on the results of this normality assessment. For each participant, the intervention effect was calculated as the change from T0 to T3 (Δ = T3 – T0), yielding ΔND_T0–T3 and ΔLPD_T0–T3 for the standard and low-protein diet groups, respectively. Comparisons between groups at T0 and at T3 were performed using either two-tailed unpaired Student’s t-test with Welch correction or a non-parametric Mann–Whitney U test, depending on data distribution. To assess within-group changes over time, paired two-tailed Student’s t-tests or Wilcoxon matched-pairs signed-rank tests were applied as appropriate. Correlations between variables were analyzed using Spearman’s rank correlation coefficient. Data are presented as means ± standard error of the mean (SEM) or as Tukey-style box-and-whisker plots, as indicated.

Statistical analyses were conducted using the R environment (version 4.0.3; https://www.r-project.org) and GraphPad Prism version 10 (GraphPad Software, San Diego, CA, USA). A two-sided p-value < 0.05 was considered statistically significant.

**Supplementary references**

1. Koppe L, Nyam E, Vivot K, et al. Urea impairs β cell glycolysis and insulin secretion in chronic kidney disease. *J Clin Invest*. 2016;126(9):3598-3612. doi:10.1172/JCI86181

2. Barba C, Benoit B, Bres E, et al. A low aromatic amino-acid diet improves renal function and prevent kidney fibrosis in mice with chronic kidney disease. *Sci Rep*. 2021;11(1):19184. doi:10.1038/s41598-021-98718-x

3. Schindelin J, Arganda-Carreras I, Frise E, et al. Fiji: an open-source platform for biological-image analysis. *Nat Methods*. 2012;9(7):676-682. doi:10.1038/nmeth.2019

4. Street JM, Souza ACP, Alvarez‐Prats A, et al. Automated quantification of renal fibrosis with Sirius Red and polarization contrast microscopy. *Physiol Rep*. 2014;2(7):e12088. doi:10.14814/phy2.12088

5. Kalantar-Zadeh K, Fouque D. Nutritional Management of Chronic Kidney Disease. *N Engl J Med*. 2017;377(18):1765-1776. doi:10.1056/NEJMra1700312

6. Maroni BJ, Steinman TI, Mitch WE. A method for estimating nitrogen intake of patients with chronic renal failure. *Kidney Int*. 1985;27(1):58-65.

7. Massy ZA, Chesnaye NC, Larabi IA, et al. The relationship between uremic toxins and symptoms in older men and women with advanced chronic kidney disease. *Clin Kidney J*. 2022;15(4):798-807. doi:10.1093/ckj/sfab262

8. Ghozlane A, Thirion F, Plaza Oñate F, et al. Accurate profiling of microbial communities for shotgun metagenomic sequencing with Meteor2. *Microbiome*. 2025;13(1):227. doi:10.1186/s40168-025-02249-w

9. Laiola M, Koppe L, Larabi A, et al. Toxic microbiome and progression of chronic kidney disease: insights from a longitudinal CKD-Microbiome Study. *Gut*. Published online June 3, 2025. doi:10.1136/gutjnl-2024-334634
